# Chemo‐Mechanical Energy Harvesters with Enhanced Intrinsic Electrochemical Capacitance in Carbon Nanotube Yarns

**DOI:** 10.1002/advs.202203767

**Published:** 2022-09-18

**Authors:** Seongjae Oh, Keon Jung Kim, Byeonghwa Goh, Chae‐Lin Park, Gyu Dong Lee, Seoyoon Shin, Seungju Lim, Eun Sung Kim, Ki Ro Yoon, Changsoon Choi, Hyun Kim, Dongseok Suh, Joonmyung Choi, Shi Hyeong Kim

**Affiliations:** ^1^ Department of Energy Science Sungkyunkwan University Suwon‐si Gyeonggi‐do 16419 Republic of Korea; ^2^ Department of Advanced Textile R&D Korea Institute of Industrial Technology Ansan‐si Gyeonggi‐do 15588 Republic of Korea; ^3^ Department of Mechanical Design Engineering Hanyang University Seoul 04763 Republic of Korea; ^4^ Department of Mechanical Engineering BK21 FOUR ERICA‐ACE Center Hanyang University Ansan‐si Gyeonggi‐do 15588 Republic of Korea; ^5^ HYU‐KITECH Joint Department Hanyang University Seoul 04763 Republic of Korea; ^6^ R&D Center A‐Tech System Co. Incheon 21312 Republic of Korea; ^7^ Department of Energy and Materials Engineering Dongguk University Seoul 04620 Republic of Korea; ^8^ Advanced Materials Division Korea Research Institute of Chemical Technology Daejeon 34114 Republic of Korea

**Keywords:** carbon nanotubes, chemo‐mechanical harvesters, energy harvesting, intrinsic electrochemical capacitance, matching impedance

## Abstract

Predicting and preventing disasters in difficult‐to‐access environments, such as oceans, requires self‐powered monitoring devices. Since the need to periodically charge and replace batteries is an economic and environmental concern, energy harvesting from external stimuli to supply electricity to batteries is increasingly being considered. Especially, in aqueous environments including electrolytes, coiled carbon nanotube (CNT) yarn harvesters have been reported as an emerging approach for converting mechanical energy into electrical energy driven by large and reversible capacitance changes under stretching and releasing. To realize enhanced harvesting performance, experimental and computational approaches to optimize structural homogeneity and electrochemical accessible area in CNT yarns to maximize intrinsic electrochemical capacitance (IEC) and stretch‐induced changes are presented here. Enhanced IEC further enables to decrease matching impedance for more energy efficient circuits with harvesters. In an ocean‐like environment with a frequency from 0.1 to 1 Hz, the proposed harvester demonstrates the highest volumetric power (1.6–10.45 mW cm^−3^) of all mechanical harvesters reported in the literature to the knowledge of the authors. Additionally, a high electrical peak power of 540 W kg^−1^ and energy conversion efficiency of 2.15% are obtained from torsional and tensile mechanical energy.

## Graphical abstract

Enhanced intrinsic electrochemical capacitance (IEC) in coiled carbon nanotube yarn harvesters is achieved by optimizing structural homogeneity and electrochemical accessible area. Stretching‐induced large variation in IEC generates the highest knowvolumetric electrical power in the ocean‐like environment when compared with other types of mechanical harvesters usable in the ocean.

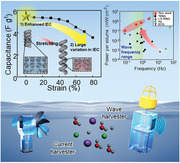

## 1 Introduction

The continuous monitoring of the environment is important to predict and prevent environmental disasters.^[^ ^1^, ^2^, ^3^ ^]^ Monitoring devices are usually located randomly across a large area where it is difficult to access and charge energy devices. To supply electrical energy to these devices, on‐site energy harvesting has been considered as a possible solution. In particular, since mechanical energy from nature, such as waves, currents, and wind, is present almost everywhere, research on mechanical harvesters using these has attracted substantial attention. For the practical application of mechanical harvesters, it is important to consider the environmental conditions depending on the specific location of the application (e.g., humidity, temperature, and mechanical frequency).

Immersed environments have been considered as a particular challenge with regards to applications of mechanical energy harvesters. Recently, two types of mechanical energy harvesters have addressed this issue.^[^ ^4^, ^5^, ^6^, ^7^, ^8^, ^9^ ^]^ The first is liquid‐solid triboelectric nanogenerators (LS‐TENGs), which generate electrical energy based on the change of the interfacial area of liquid on the solid surfaces of the LS‐TENGs. The LS‐TENGs generate high electrical energy and enable operation of low‐power electrical devices, such as light emitting diodes (LED) and sensors, using liquid motion (< 2 Hz).^[^ ^6^, ^7^, ^8^, ^10^ ^]^ However, the LS‐TENGs generate higher power in deionized water than electrolytes because existing ions prohibit their electricity generation.^[^ ^7^, ^8^, ^11^, ^12^ ^]^ Thus, it is more suitable to use LS‐TENG in environments with low ion concentrations, such as rivers and waterfalls. The second is chemo‐mechanical energy harvesters consisting of electrodes and electrolytes.^[^ ^9^, ^13^, ^14^, ^15^, ^16^ ^]^ Electrodes have an electrochemically accessible area within electrolytes, and this area forms an intrinsic electrochemical capacitance (IEC). When the IEC changes with external mechanical force, chemo‐mechanical energy harvesters generate electrical energy, thus, maximization of IEC variation can play a key role to enhance performance.^[^ ^9^, ^14^, ^15^, ^16^ ^]^ Since 2017, a new type of coiled carbon nanotube (CNT) yarn chemo‐mechanical energy harvesters named “Twistron” have been reported.^[^ ^14^, ^16^, ^17^ ^]^ The Twistron, which is the coiled CNT yarn, is fabricated by twisting multi‐walled carbon nanotube (MWCNT) sheets.^[^ ^18^, ^19^ ^]^ Stretching the coiled CNT yarn induces a substantial change in the IEC and generates the highest peak power or frequency‐normalized peak power between a few Hz and 600 Hz, compared to other types of chemo‐mechanical harvesters. However, so far, it has been reported that the IEC inevitably decreases during conventional twist spinning, preventing the further performance enhancement of Twistron‐based harvesters.^[^ ^14^, ^16^ ^]^

In this study, we demonstrated a coiled CNT yarn with enhanced IEC and performance through novel twist spinning approaches. Our optimized coiled CNT yarn simultaneously provided a large IEC (5.6 F g**^−^** ^1^) and large IEC variations (35%). Using this novel structure in in coiled CNT yarn, our chemo‐mechanical harvester delivered 2.6 times the open circuit voltage (OCV, 270 mV), 4 times the peak power (540 W kg**^−^** ^1^), and twice the efficiency (2.15%) of prior Twistron reported in 2017.^[^ ^14^ ^]^ In particular, it achieved the highest volumetric power at 0.1–1 Hz compared to other chemo‐mechanical harvesters thus, demonstrated promising potential for oceanic harvester applications. Overview of this work is illustrated in Figure S1 (Supporting Information).

## 2 Results and Discussion

### 2.1 Fabrication and Working Mechanism of L‐coiled yarn harvesters

**Figure 1:**
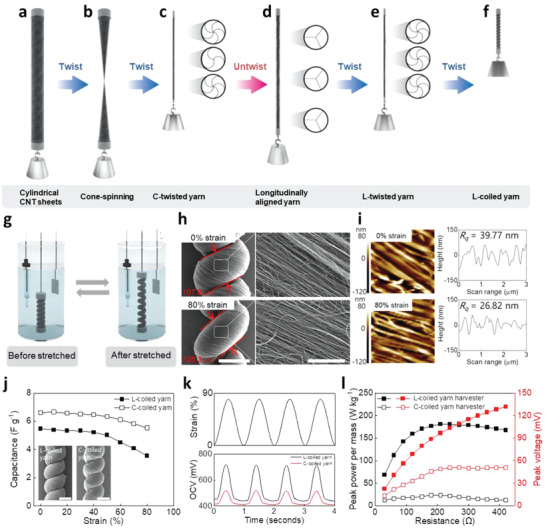
L‐Coiled yarn harvester fabrication, morphology, and electrochemical performance in 0.1 M HCl. a–f) Illustration of the L‐coiled yarn fabrication process. a) Cylindrical CNT sheets, b) cone‐spinning, c) C‐twisted yarn, d) LAY, e) L‐twisted yarn, and f) L‐coiled yarn. g) Configuration of the coiled CNT yarn‐based harvester (reference electrode: Ag/AgCl, counter electrode: Pt mesh/MWCNT, bucky paper). h) SEM images showing L‐coiled yarn at 0% and 80% strain (scale bar: 100 µm, scale bar: 5 µm). i) AFM images and graphs showing the height differences of the surface in L‐coiled yarn at 0% and 80% strain in the range of 3 × 3 µm. j) Gravimetric capacitance induced by tensile strain and inset showing SEM images of L‐coiled yarn (left) and C‐coiled yarn (right). k) Real‐time *OCV* for four cycles of L‐ and C‐coiled yarn during stretching to 80%. l) Peak power per mass (red) and peak voltage (black) induced by the load resistance for the L‐ and C‐coiled yarn harvesters when stretched to 80% at 1 Hz.

**Figure** **1**a–f shows the novel twist spinning process of coiled CNT yarn to enhance IEC variation and electrical performance. The overall process is named longitudinally aligned yarn (LAY) spinning. To produce LAY‐spun yarn, MWCNT sheets that were drawn from MWCNT forests were assembled into ≈4 cm wide, ≈30 cm long sheet stacks wherein the MWCNTs were aligned along the sheet length direction.^[^ ^19^ ^]^ The MWCNT sheets were manually rolled into a cylindrical shape at a certain load to prepare cylindrical CNT sheets (Figure 1a). A twist was inserted from the central axis of this cylinder, and this process was called cone‐spinning (Figure 1b).^[^ ^14^ ^]^ The sheet stack deformed into two cones during this process, forming a densely packed yarn between these cones, termed C‐twisted yarn (Figure 1c; and Figure S2, Supporting Information). These processes (Figure 1a–c) inserted a twist while prohibiting the rotation of the bottom end of the cylindrical sheets. The LAY (Figure 1d, Figure S3, Supporting Information) was prepared by removing the prohibition of rotation at the bottom end with same load with above process. After self‐untwisting, second twist was inserted again into the LAY while prohibiting rotation at its bottom part. After the untwisting, during the second twisting process, L‐twisted yarn was fabricated (Figure 1e), then L‐coiled yarn was produced subsequently with further twisting (Figure 1f; and Figure S4, Supporting Information). It should be emphasized that the LAY‐spinning, the processes from C‐twisted yarn to L‐twisted yarn, provided outstanding structural uniformity. The C‐twisted yarn made from the cone‐spinning method provided a cross‐sectionally homogeneous structure attributed to the symmetrical cone shape.^[^ ^14^ ^]^ However, the cross‐sectional homogeneity did not extend along the entire length of the C‐twisted yarn. As shown in Figure S5 (Supporting Information), during the cone‐spinning process for C‐twisted yarn, angles between twist completed yarn and remaining cylindrical sheet can be significantly different along the longitudinal direction. This angle distribution causes stress gradient and structural inhomogeneity indicated by large variation in bias angle (24°–32°) and twist numbers along the length direction of the C‐twisted yarns. To extend the cross‐sectional homogeneity into the entire length of coiled yarn, the untwisting‐twisting process, LAY‐spinning, was introduced. The LAY‐spinning enabled to minimize stress gradient along the entire length, thus, provided an excellent structural homogeneity with uniform bias angle variation in coils (31°–33°), as shown in Figure S6 (Supporting Information). Then, further coiling process from this uniform L‐twisted yarn enables homogeneous twist insertion for L‐coiled yarn (Figure S7, Supporting Information). To optimize the LAY‐spinning process, repeating untwisting‐twisting cycle effect was further investigated. A negligible slight structural variation in L‐coiled yarns with repeating LAY‐spinning suggests that only one cycle of untwisting‐twisting could be enough to achieve homogeneity in L‐coiled yarns (Figure S8 and Table S1, Supporting Information). As a result, it was noticed that electrical power retention ratio was well maintained along the entire length of the L‐coiled yarn (Figure S9, Supporting Information).

Figure 1g shows three‐electrode system for evaluating the electrochemical performance of the coiled CNT yarn harvesters. Details of the electrochemical tests are described in the experimental part of the Supporting Information. This configuration briefly illustrated the operation mechanism of the coiled yarn harvesters with mechanical stretching and releasing deformation. All strains on the coiled CNT yarn were assessed within a range that could be operated stably without failure. When stretching the coiled CNT yarn, the internal twist density increases, thus CNT yarn's volumetric density also increases.^[^ ^14^, ^16^, ^17^ ^]^ This change resulted in a decrease in capacitance (*C*), thereby producing a voltage (*V*) increase. In other words, the coiled CNT yarn converted mechanical energy into the decrement of IEC without external bias voltage. Based on this mechanism, coiled CNT yarn generated electrical energy (Figure S8, Supporting Information) and the variation in IEC which can be maximized further to enhance performance.^[^ ^14^, ^16^, ^17^ ^]^

Figure 1h,i shows changes in the surface morphology of the L‐coiled yarn with 0% (top) and 80% strain (bottom). As shown in the scanning electron microscopy (SEM) images, the diameter of L‐coiled yarn was found to decrease by 2.5 µm at 80% strain, which is twice higher than C‐coiled yarn case (Figure S9, Supporting Information). Furthermore, the compressive stress from stretching induced the densification of the CNTs coiled yarns. Under mechanical stretching up to 80%, both L‐ and C‐coiled yarns were densely packed and resulted in smaller standard deviation of surface height (*R*_q_) than initial 0% strain. As shown in the atomic force microscopy (AFM) images, *R*_q_ at 0% strain in L‐ and C‐coiled yarn was 39.77 and 68.4 nm, respectively, indicating that the L‐coiled yarn had a smoother surface (i.e., more uniform densification) than the C‐coiled yarn. When stretched, importantly, L‐coiled yarns provide higher *R*_q_ changing ratio (32%, Figure S10, Supporting Information) than C‐coiled yarn (17%, Figure S11, Supporting Information). When this variation occurred in electrolytes, electrochemically accessible area could be also decreased during stretching, thus, induced subsequent changes in IEC. Through the larger morphology variation in L‐coiled yarn, variation of electrochemical properties can be greater.

To compare resulting electrochemical properties driven from the improved fabrication methods and structural homogeneities, equivalent resisting stress (1.18 MPa) was applied to both of L‐ and C‐coiled yarns during twist processes. As shown in Figure 1j, L‐ and C‐coiled yarns exhibit similar spring index (L‐coiled yarn: 0.65; C‐coiled yarn: 0.64) and yarn diameter (L‐coiled yarn: 104.3 µm; C‐coiled yarn: 106.9 µm). Even both yarns were prepared to have similarity in microscale structures, it should be emphasized that electrochemical properties can be significantly different due to the nanoscale structural properties. Capacitances of L‐ and C‐coiled yarn were 5.47 and 6.61 F g^−1^ at 0% strain, and 3.55 and 5.53 F g^−1^ at 80% strain, respectively. The L‐coiled yarn had twice difference in capacitance changes (*∆C*) before and after stretching based on the larger changes in nanoscale surface morphology. With the outstanding change in IEC, L‐coiled yarn generated the higher variation in OCV (∆V) and short‐circuit‐current than C‐coiled yarn (Figure 1k; and Figure S14, Supporting Information). As a result, the L‐coiled yarn harvester generated an eight times higher electrical peak power of 181 W kg^−1^ than the C‐coiled yarn harvester (Figure 1l).

### 2.2 Charcterization of the L‐coiled yarn harvesters

**Figure 2:**
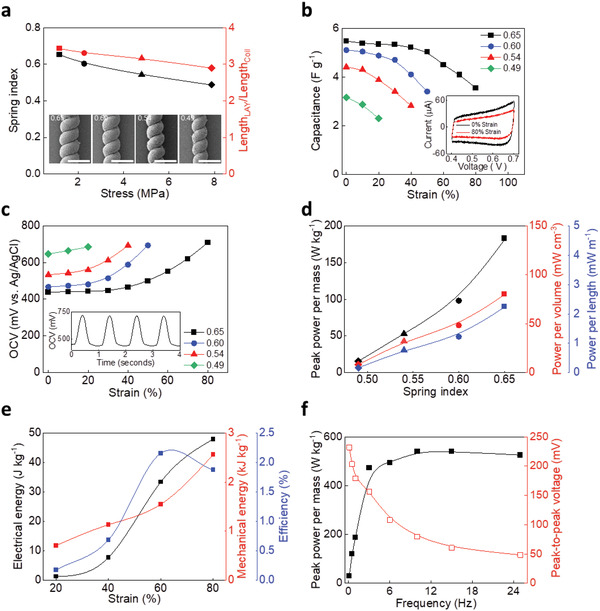
Characterization of L‐coiled yarn. a–d: Squares, circles, triangles, and diamonds indicate a spring index of 0.65, 0.60, 0.54, and 0.49, respectively. a) Stress dependence of the spring index (black) and *Length*_LAY_ */Length*_Coil_ (red). Inset showing the SEM images at spring indexes of 0.65, 0.60, 0.54, and 0.49 (scale bar: 200 µm). b) Gravimetric capacitance (inset showing cyclic voltammetry curve at 0% and 80% strain), c) *OCV* versus strain depending on the spring index of L‐coiled yarns, with corresponding raw data of (b,c) shown in insets. d) Normalized peak power to mass (black), volume (red), and length (blue) of L‐coiled yarn harvesters versus spring indexes under matching impedance, with matching impedances plotted in the inset. e) Frequency dependence of peak power per mass (black) and peak‐to‐peak *OCV* (red) of L‐coiled yarn harvester with a spring index of 0.65. f) Electrical energy (black), the required mechanical energy (red), and energy conversion efficiency (blue) of L‐coiled yarn harvester.

To further optimize L‐coiled yarn system, a systematic study was conducted by controlling spring indexes of 0.65, 0.60, 0.54, and 0.49 by applying stress of 1.18, 2.27, 4.8, and 7.8 MPa, respectively, during the twisting process from LAY to L‐coiled yarns (**Figure** **2**a). As the spring index decreased from 0.65 to 0.49, the yarn diameter was decreased from 107.6 to 97.2 µm, as well as the *Length*_LAY_/*Length*_Coil_ also reduced from 3.4 to 2.9. The *Length*_LAY_ */Length*_Coil_ is defined as the length of the LAY divided by the length of the L‐coiled yarn and represents the amount of LAY stored in the same coil length. In short, the L‐coiled yarn with the largest spring index had the lowest yarn density and largest amount of CNTs. Figures 2b,c display the capacitances and OCVs during stretch of the L‐coiled yarns as a function of the spring index. In order of increasing spring index, the capacitances at 0% strain of L‐coiled yarns were 3.16, 4.41, 5.10, and 5.47 F g^−1^ and the *∆C* were 0.8, 1.6, 1.7, and 2 F g^−1^ (Figure 2b). The L‐coiled yarn with a spring index of 0.65 provided the largest change in capacitance (35%) at 80% strain, thus, enabled 270 mV of ∆V, as measured with the real time OCV monitoring during mechanical deformation (Figure 2c; and Figure S15, Supporting Information). Thus, electrochemical properties of the harvesters can be more dramatically shifted with larger spring index (i.e., with lower yarn density and higher amount CNTs). In consequences, output electrical performance of the harvesters also improved. The electrical power of L‐coiled yarn harvesters with a spring index of 0.65 was measured as 181 W kg^−1^, 80.4 kW m^−3^, and 2.25 mW m^−1^ at 1 Hz (Figure 2d; and Figure S16, Supporting Information).

As discussed above, the improved electrical power of an L‐coiled yarn harvesters resulted from both increased initial IEC and changes in IEC under stretching. To achieve this, the fabrication of LAY is essential because it allows homogeneous twist insertion with low applied stress. The decrement in IEC can be prevented while the LAY is densified by twist insertion using low stress to spin L‐coiled yarn with a high spring index. Therefore, it is possible to construct a larger electrochemical accessible area in L‐coiled yarns to improve energy harvesting performances. The mechanical‐to‐electrical energy conversion efficiency of the L‐coiled yarn harvester was obtained as 2.15% at 60% stretching and 1 Hz (Figure 2e; and Figure S17, Supporting Information). This result was twice that of previously reported coiled CNT yarn harvesters.^[^ ^14^ ^]^

The L‐coiled yarn delivered a maximum peak power per mass of 540 W kg^−1^ under 240 Ω at 10 Hz, and 97% of this electrical power was also maintained at a high frequency of 25 Hz (Figure 2f). It is important to identify the factors affecting matching impedance to understand the frequency‐dependent performance of coiled yarn harvesters, and that it is supported by the simple R‐C model. In the harvester circuit

(1) $$Z_{\mathrm{harvester}}=R_{\mathrm{internal}}+1/jwC$$

where *Z* is impedance, *R* is resistance, j is the root −1, *w* is angular frequency, and *C* is capacitance.^[^ ^14^ ^]^ The peak power per mass sharply increased within below 10 Hz frequency. This means the matching impedance is more dominated by the double‐layer capacitance term (1/jw*C*) in the above formula than the internal resistance of the yarn harvester. Then, the double‐layer capacitance dominance was decreased above 10 Hz frequency and the matching impedance was dominated by the internal resistance of the L‐coiled yarn. As the internal resistance was constant, peak power per mass became saturated at 540 W kg^−1^ above 10 Hz.

To summarize, at a frequency below 10 Hz, the improved double‐layer capacitance in the L‐coiled yarn enables lowering the matching impedance. Our L‐coiled yarns provide improved capacitance owing to two significant characteristics: i) relatively low density with lower applied coiling stress and ii) high length storage capacity expressed as *Length*_LAY_ */Length*_Coil_. Thus, L‐coiled yarn can offer a larger electrochemical accessible area in the electrolytes. This improved capacitance effectively reduced the matching impedance from 390 to 210 Ω with spring index from 0.49 to 0.65 (Figure S18, Supporting Information). Reducing the matching impedance is of significant interest in the mechanical harversters to improve the energy transfer efficiency,^[^ ^20^ ^]^ thus, we believe our L‐coiled yarn harvester was able to deliver high energy transfer efficiency, as described above. In addition, to further understand performances of L‐coiled yarn harvesters in various potential environments, extended conditions have been investigated with several types of electrolytes (acidic, neutral, and basic), electrolyte concentration (0.001–5 m), and temperatures (0–30 °C). Briefly, 0.1 m of acidic HCl demonstrated highest and stable electrical output performances, as shown in Figure S19 (Supporting Information) and associated detailed explanation.^[^ ^21^, ^22^ ^]^

### 2.3 Structural Analysis

So far, enhanced harvesting performance has been demonstrated through experimental approaches. It should be noted that these results originated from the large structural variation in nanoscale of L‐coiled yarn. In particular, the interaction between the MWCNTs and surrounding electrolytes provided an electrochemical accessible area to electrochemical double layer (EDL), which induced by the adsorption and desorption of ions from electrolytes.^[^ ^23^ ^]^ Variations in the ion distribution in the EDL are the basic principle for the generation of electrical energy in coiled CNT yarn harvesters,^[^ ^14^, ^16^, ^17^ ^]^ which is driven by the structural variation of the L‐coiled yarn. Therefore, geometrical analysis was conducted to understand the structural changes of the L‐coiled yarn during mechanical deformation.

**Figure 3:**
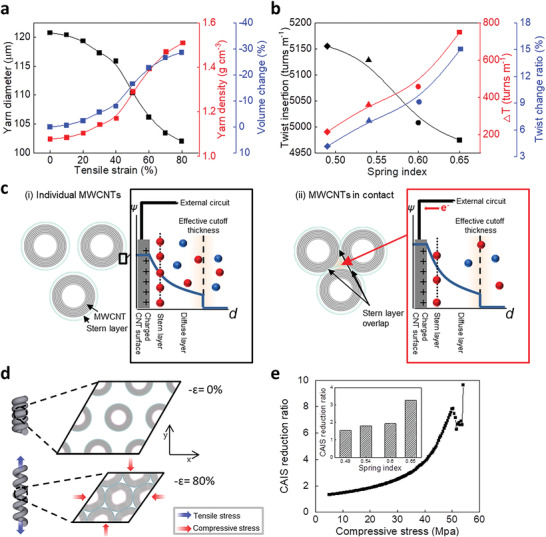
Understanding the relationship between the inner structure and IEC change of coiled CNT yarn. a) Tensile strain dependence of the yarn diameter (black), yarn density (red), and volume change (blue) in L‐coiled yarn with a spring index of 0.65 during stretching 80%. b) Twist insertion (black), *∆T* (red), and twist change ratio (blue) of L‐coiled yarns with spring indexes of 0.49, 0.54, 0.60, and 0.65. c) Conceptual illustration showing the change in the electrochemical double layer. The potential changes near the surface of MWCNTs (*ψ*) were depicted (i) when each MWCNT was far apart and (ii) when MWCNTs were adjacent to each other. The red and blue spheres represent negative and positive charges, respectively. d) The mechanism by which the tension of the L‐coiled yarn exerts a compressive strain on the cross‐sectional microstructure. As the compressive strain reaches 40%, the adjacent MWCNTs come into contact with each other, and compressive stress is generated. e) The CAIS reduction ratio under radial compressive stress. The equivalent value according to the spring index condition considered in the experiment is included as an inset.

The changes in yarn diameter induced by the strain results additional twists (*∆T*) for maintaining the twist per fiber length of the L‐coiled yarn (see the Experimental Section for detailed equations). The *∆T* densified the L‐coiled yarn, as shown in **Figure** **3**a,b. The L‐coiled yarn with a greater spring index is more densified at a higher *∆T*, despite having a lower initial yarn density. The proportional relation of densification by *∆T* and the variation in IEC indicates that the L‐coiled yarn with a larger spring index delivers a larger variation in IEC. The microscopic perspective of the relationship was also supported through molecular dynamics (MD) simulation.

The change in yarn diameter, yarn density, and volume change of L‐coiled yarn with various spring indexes are shown in Figure 3a; and Figures S20–S24 (Supporting Information). The initial yarn diameter was 120 µm and decreased by 18.74 µm by 80% stretching deformation. Therefore, the yarn density increased from 1.08 to 1.51 g cm^−3^ and the volume was contracted to 28%. These physical properties were investigated for the L‐coiled yarn with other spring indices. The yarn density of L‐coiled yarn with spring indices of 0.60, 0.54, and 0.49 decreased by 0.40, 0.28, and 0.14 g cm^−3^, respectively. The electrochemical properties depend on two representative twisting properties in L‐coiled yarn. One is initial twist insertion from LAY to L‐coiled yarn, which determines the initial IEC. The other is an additional twist that determines the decrease in IEC induced by tensile strain. These were investigated using a rotation counting machine and a formula for calculating *∆T* by strain. In order of decreasing spring index, in L‐coiled yarn there were 4975, 5008, 5128, and 5155 turns/m and the *∆T* was 748, 457, 360, and 214 turns m^−1^, respectively (Figure 3b). The L‐coiled yarn with a large spring index showed a large initial IEC and a large variation in IEC owing to the exceptional twist properties.

The harvesting performance was improved with more twist insertion due to the greater *∆T*, which increases the yarn density change with stretching.^[^ ^17^ ^]^ However, L‐coiled yarn with a spring index of 0.65 was fabricated with the lowest twists (4975 turns m^−1^) and provided the greatest *∆T* (748 turns m^−1^). From these results, we can suggest a model for the initial inner structure composed of CNTs and its variations. The L‐coiled yarn with a larger spring index had greater spacing between the individual CNTs without strain, with greater density change on the addition of strain. Therefore, the L‐coiled yarn with a large spring index had a larger initial IEC and larger decrease in IEC. This tendency between twist insertion and *∆T* was exceptional compared with other reported coiled CNT yarn^[^ ^17^ ^]^ and our L‐coiled yarn could effectively harvest the mechanical energy applied.

The decrement of IEC according to the increase in areal density for the cross‐section of L‐coiled yarn is presented as a scheme in Figure 3c. It was assumed that the electrochemical double‐layer structure formed on the surface of MWCNTs followed the model proposed by Gouy–Chapman.^[^ ^24^ ^]^ The MWCNT surfaces are hole‐injected in the HCl electrolyte, and Cl^‐^ ions screened on the solid‐liquid surfaces to compensate electrical charge for thermodynamic equilibrium (Figure 3c(i)). When stretching the harvester, the compressive force induces desorption of Cl^‐^ ions, and the potential of the MWCNTs is increased by reducing the screened anions (Figure 3c(ii)). Thus, electrons move to the MWCNTs by increasing the potential. After releasing the harvester, the MWCNTs apart from each other by decreasing compressive force, and immersing Cl^−^ ions into the MWCNTs induces that the electrons flow out through the external circuit. The change in the interstitial sites formed by the spacing between MWCNTs is thus believed to be an important factor. If the distances between the MWCNTs are extremely close, a further reduction in IEC is expected with the radial buckling of the MWCNTs.

To comprehend the effect of the areal density of L‐coiled yarn on IEC at a nanoscale, all‐atom MD simulations were conducted. The macroscopic stretching applied to the coiled CNT yarns acted as an in‐plane compressive strain that reduced the spacing between the MWCNTs, as shown in Figure 3d. In this regard, the in‐plane stress of MWCNT yarn and the corresponding change in the cross‐sectional area of interstitial sites (CAIS) during the biaxial compression were calculated (Figure S25, Supporting Information). Based on the yarn diameter change and initial yarn capacitance of 5 F g^−1^ obtained from the experiment, the equivalent CAIS reduction ratio for each spring index was also calculated.

Figure 3e shows the relationship between the CAIS reduction rate and compressive stress. The reduction rate of CAIS increased exponentially and then decreased irregularly. The highly nonlinear behavior was induced by the elastic collapse of interstitial sites as MWCNTs began to contact each other and then experienced radial buckling (Figure S26, Supporting Information). However, the experimental results suggest that the compressive stress was maintained at a low level (< 37 MPa) under all conditions (not shown in the figure). Therefore, the CAIS reduction ratio appeared to increase monotonically as the spring index increased, in agreement with experimental findings. In addition, when the CAIS reduction ratio was calculated from the change in yarn diameter, the present L‐coiled yarn had a value of 3.28, whereas it reached 7.8 in the ideally dispersed state of MWCNTs. Considering that the reduction rate of CAIS is closely related to the amount of change in IEC, the difference suggests that the L‐coiled yarn has potential for further performance enhancement.

### 2.4 Demonstration and Performance as Oceanic Harvesters

**Figure 4:**
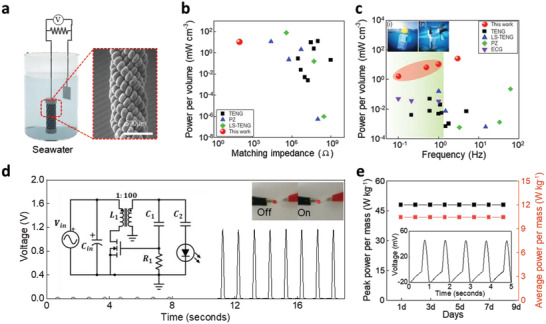
Characterizing application of the L‐coiled yarn harvester and comparisons with other mechanical harvesters used in the ocean. a) Schematic and SEM image of an 8‐plied L‐coiled yarn harvester. (scale bar: 500 µm). b) Matching impedance dependence of power per volume for various mechanical energy harvesters, such as TENGs, PZs, and LS‐TENGs. c) Frequency dependence of power per volume for TENGs, PZs, ECGs, and LS‐TENGs. The green area indicating less than 1 Hz and application illustrations of L‐coiled yarn harvester using (i) ocean waves and (ii) currents is shown in the inset. (Detailed operation processes are represented in Figure S29). d) Voltage versus time graph (inset showing the harvesting circuit to amplify the voltage and photograph of the red LED). e) Peak power per mass (black) and average power per mass (red) of 8‐plied L‐coiled yarn harvester for 8 days and typical *OCV* variation by tensile strain are shown in the inset.

To demonstrate potential applications with our harvesters, **Figure** **4**a; and Figure S27 (Supporting Information) show the nulti‐plied, 4‐ and 8‐plied, L‐coiled yarn with 3.46 mg of resisting weight. It generated a peak power per mass of 77 W kg^−1^ at 90 Ω in seawater collected from the Gyeonpo Sea off South Korea (Figure S28a, Supporting Information) and maintained an electrical peak power of 48 W kg^−1^ after training for 4 days (Figure S28b, Supporting Information). The scale‐up of the L‐coiled yarn harvester provided not only an increase in peak power but also decrease in matching impedance, similar to parallel connections. The matching impedance‐dependent volumetric power of an 8‐plied L‐coiled yarn harvester was compared with that of various mechanical harvesters, such as TENGs,^[^ ^25^, ^26^, ^27^, ^28^, ^29^, ^30^, ^31^, ^32^ ^]^ piezoelectric nanogenerators (PZs)^[^ ^33^, ^34^, ^35^, ^36^ ^]^, and LS‐TENGs^[^ ^37^, ^38^, ^39^ ^]^ (Figure 4b). To compare the volumetric power with other types of mechanical harvesters, we normalized the generated electrical power using the total unit volume of the harvesting system containing both coiled CNT yarns and Pt mesh. Our L‐coiled yarn harvester delivered considerable volumetric power (10.45 mW cm^−3^) at a remarkably low matching impedance (90 Ω). This enables our 8‐plied L‐coiled yarn harvester could effectively improve the energy transfer efficiency with reduced matching impedance.^[^ ^20^ ^]^ The frequency‐dependent volumetric power was also compared with that of other mechanical energy harvesters usable in the ocean including TENGs,^[^ ^40^, ^41^, ^42^, ^43^, ^44^, ^45^, ^46^, ^47^, ^48^, ^49^ ^]^ PZs,^[^ ^36^, ^50^, ^51^ ^]^ electrochemical generators (ECGs),^[^ ^15^ ^]^ and LS‐TENGs.^[^ ^37^, ^52^, ^53^ ^]^ Considering the low frequency environment of the ocean (< 1 Hz), the collected frequency was typically below 100 Hz. In particular, the frequency range of an ocean wave of less than 1 Hz (green area) should be highlighted because our L‐coiled yarn harvester reached the highest volumetric power (1.6–10.45 mW cm^−3^) from 0.1 to 1 Hz, as shown in Figure 4c. To demonstrate operation of L‐coiled yarn harvester in the real ocean, the harvester was installed in the ocean as illustrated in Figure 4c inset (i) and Figure S29a (Supporting Information). A 5.28 mg of L‐coiled yarn harvester was stretched with the raise of the connected buoy, then generated electrical power in response to real waveform from the Bangameori Sea of South Korea. This harvester delivers the gravimetric and absolute average power output of 2.46 W kg^−1^ and 13 µW, respectively, with wave frequencies of 0.6–1.1 Hz (Figure S30, Supporting Information).

The 8‐plied L‐coiled yarn harvester was further integrated into an energy harvesting system, including a boosting circuit and red LED, as shown in Figure 4d. It generated 23 mV at 13 Ω in the input port, which was amplified to 1.2 V from the boosting circuit for directly illuminating the LED. The low matching impedance allowed sufficient voltage generation to transfer to the boosting circuit at a low resistance of the input port. The LED was directly turned on at every peak of the amplified voltage, as shown in the optical images in Figure 4d. L‐coiled yarn harvester showed stable cycle durability for 10 000 cycles, as shown in Figure S31, thus, enabled stable performances for several days. The inset of Figure 4e shows the typical voltage variation on stretching the harvester for five cycles at 1 Hz. The 8‐plied L‐coiled yarn harvester steadily provided a peak voltage, peak power, and average power of 46 mV, 48 W kg^−1^, and 10 W kg^−1^, respectively. This performance was maintained for 8 days as shown in Figure 4e; and Figure S32 (Supporting Information).

## 3 Conclusion

To sum up, we demonstrated the newly suggested LAY‐spinning method and L‐coiled yarn harvesters which enable a high electrical power of 540 W kg^−1^ and high electrical energy generation efficiency of 2.15%, thus being promising as a chemo‐mechanical harvester. This approach constructed an electrochemically effective structures using the coiled CNT yarn, which allowed the L‐coiled yarn to have a large IEC and large IEC changes induced by tensile and torsional mechanical energy. These are exceptional properties for the coiled CNT yarn harvester because a large IEC and large IEC changes are often incompatible. The L‐coiled yarn harvester provided the highest volumetric power (1.6–10.45 mW cm^−3^) for a range of ocean wave frequency (0.1–1 Hz) within seawater environment compared to other previously reported harvesters. To realize practical application of our L‐coiled yarn harvesters in actual ocean, a variety of complex environmental factors should be considered including ionic concentration, pH, wave height, wave frequency. We believe presented approaches including the LAY spinning process, related electrical circuit integration, and lowering matching impedance could be a guidance for future studies.

## Conflict of Interest

The authors declare no conflict of interest.

## Supporting information

Supporting Information

## Data Availability

The data that support the findings of this study are available from the corresponding author upon reasonable request.
