# Supplementary material for: Chemo‐Mechanical Energy Harvesters with Enhanced Intrinsic Electrochemical Capacitance in Carbon Nanotube Yarns
Source: Adv Sci (Weinh). 2022 Sep 18;9(32):2203767. doi: 10.1002/advs.202203767 (PMC9661839; doi:10.1002/advs.202203767)
Supplement: Supplementary file 1 — Supporting Information [file ADVS-9-2203767-s001.pdf]

Chemo-mechanical energy harvesters with enhanced intrinsic electrochemical capacitance in carbon nanotube yarns

Seongjae Oh[1,2+], Keon Jung Kim[2+], Byeonghwa Goh[3,4], Chae-Lin Park[5], Gyu Dong Lee[2], Seoyoon Shin[2], Seungju Lim[2], Eun Sung Kim[6], Ki Ro Yoon[2], Changsoon Choi[7], Hyun Kim[8], Dongseok Suh[1], Joonmyung Choi[3,4,5*], Shi Hyeong Kim[2,5*]

[1]*Department of Energy Science, Sungkyunkwan University, Suwon-si, Gyeonggi-do 16419, Republic of Korea*

[2]*Department of Advanced Textile R&D, Korea Institute of Industrial Technology, Ansan-si, Gyeonggi-do 15588, Republic of Korea*

[3]*Department of Mechanical Design Engineering, Hanyang University, Seoul 04763, Republic of Korea*

[4]*Department of Mechanical Engineering, BK21 FOUR ERICA-ACE Center, Hanyang University, Ansan-si, Gyeonggi-do 15588, Republic of Korea*

[5]*HYU-KITECH Joint Department, Hanyang University, Seoul 04763, Republic of Korea*

[6]*R&D Center, A-Tech System Co., Incheon 21312, Republic of Korea*

[7]*Department of Energy and Materials Engineering, Dongguk University, Seoul 04620, Republic of Korea*

[8]*Advanced Materials Division, Korea Research Institute of Chemical Technology, Daejeon 34114, Republic of Korea*

*Correspondence and requests for materials should be addressed to S. H. K. (shk@kitech.re.kr) and J. M. C. (joonchoi@hanyang.ac.kr)

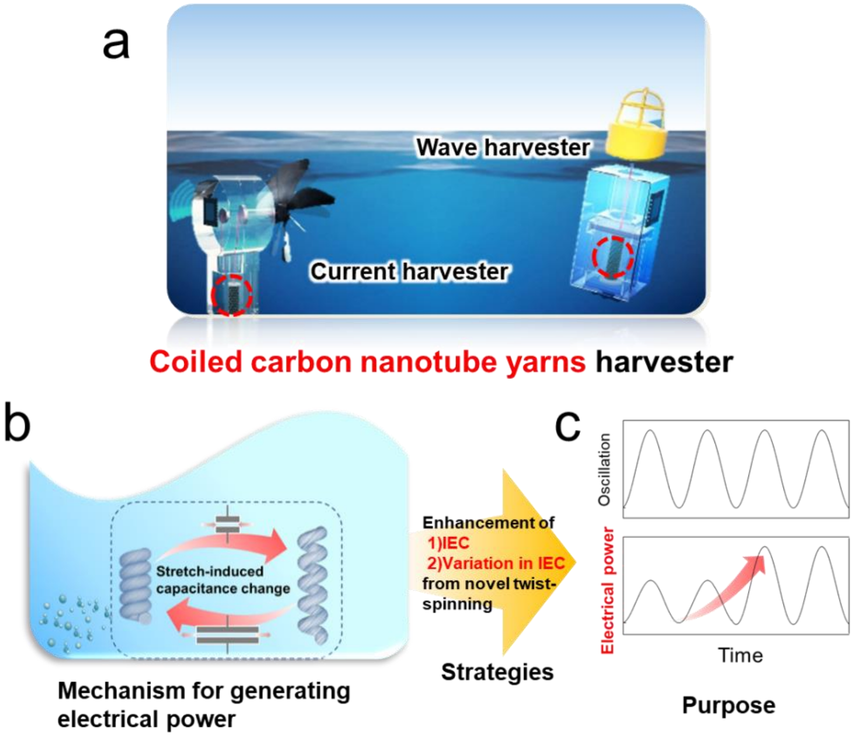

Supplementary Figure S1. Schematic of the proposed coiled carbon nanotube yarn chemo-mechanical energy harvesters showing a) future application, b) mechanism for generating electrical power, and c) purpose of this work. a) Schematic of chemo-mechanical energy harvesters showing an integrated system with electrical devices in the ocean, one of the applicable environments. b) Mechanism to generate electrical energy in coiled carbon nanotube yarn harvester. In the electrolytes, a coiled carbon nanotube yarn with intrinsic electrochemical capacitance (IEC) stores electrical energy as a supercapacitor. Using the stretching and releasing deformation in coiled carbon nanotube yarns, the electrochemical accessible area forming IEC can be reversibly decreased and recovered, thus, generate electrical energy. From enhanced IEC and IEC variation in coiled carbon nanotube yarns, we demonstrate the chemo-mechanical energy harvester with improved output electrical power as shown in c).

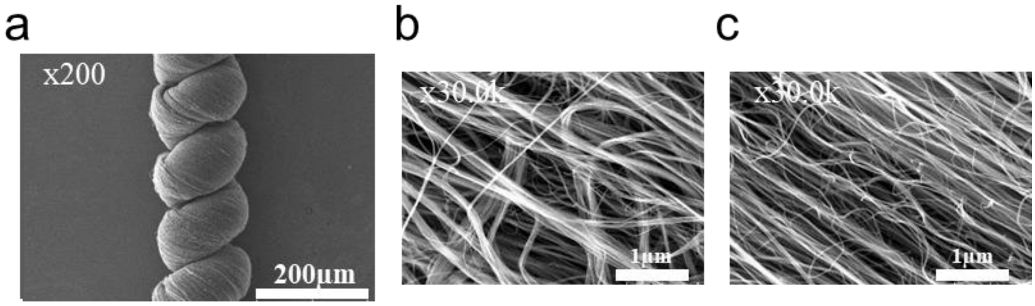

Supplementary Figure S2. Scanning electron microscopy (SEM) images of C-coiled yarn.

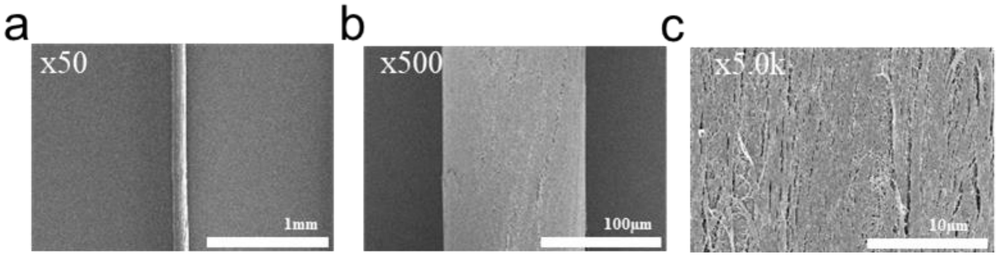

Supplementary Figure S3. SEM images of longitudinally aligned yarn.

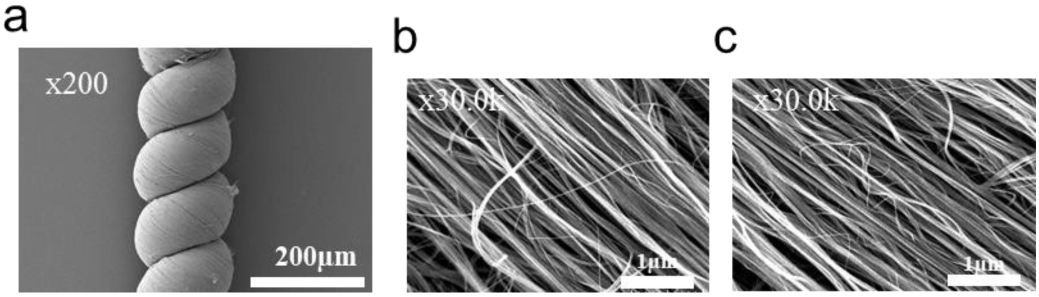

Supplementary Figure S4. SEM images of L-coiled yarn.

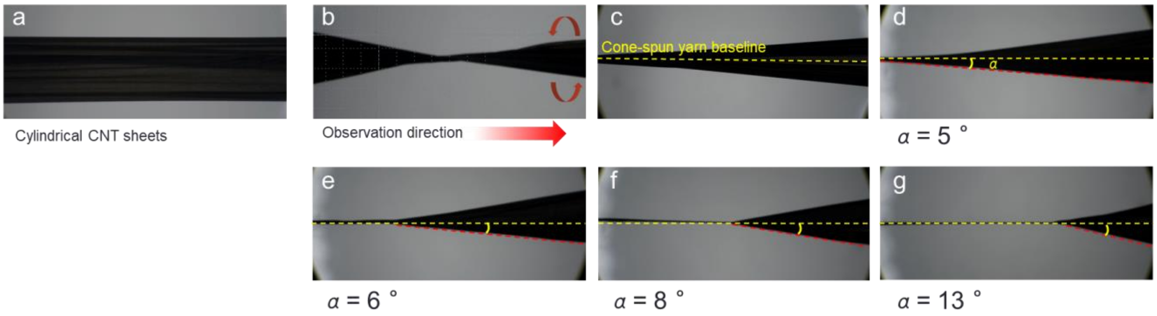

Supplementary Figure S5. a-f) Optical images showing fabrication process from cylindrical CNT sheets to cone-spinning. The α indicates angle between cone-spun yarn and cone-shaped CNT sheets.

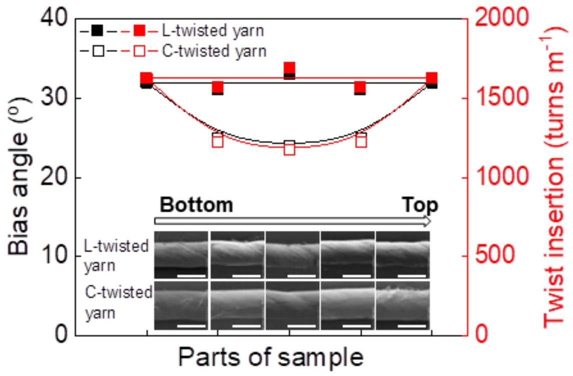

Supplementary Figure S6. Bias angles (black) and twist insertion (red) of the L- and C-twisted yarn from bottom to top. Inset shows SEM images of L- (upper) and C-twisted yarn (lower) (scale bar: 100 μm). The bias angle is alternated to twist insertion (turns m^{-1}) by the following equation: $\alpha_f = tan^{-1}(2\pi rT)$, where α_f is the bias angle, r is the radius of twisted yarn, and T is the number of turns inserted.

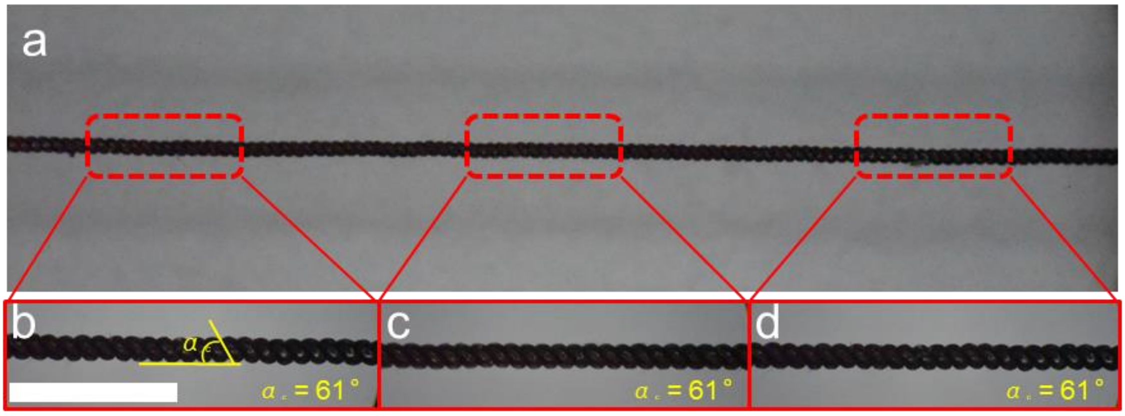

Supplementary Figure S7. Optical images showing an L-coiled yarn electrode. a) Overall L-coiled yarn electrode; b–d) parts of the L-coiled yarn electrode in detail (scale bar: 1 mm). The angle between the direction of the coiled carbon nanotube (CNT) yarn and individual coils is indicated as the coil bias angle α.

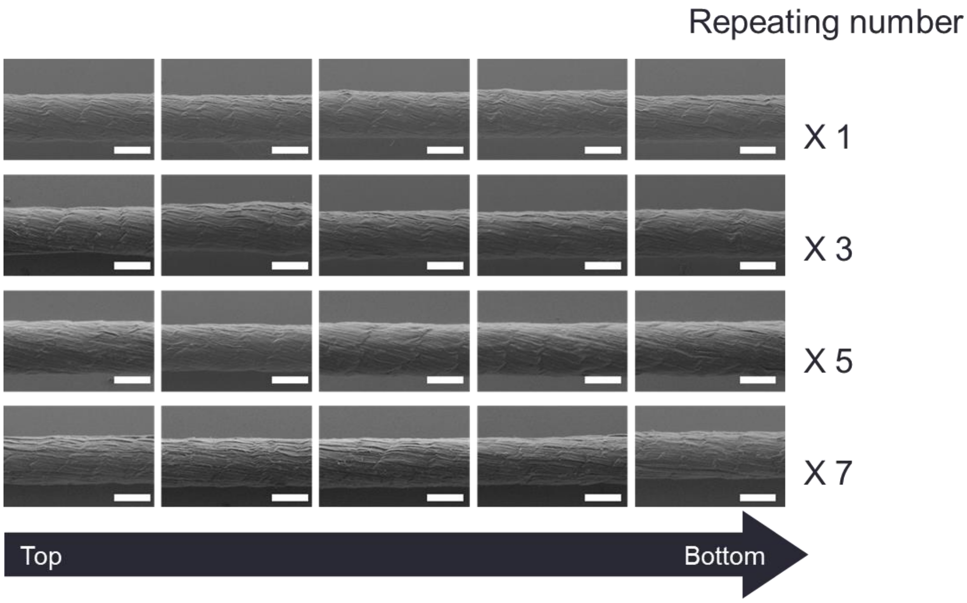

Supplementary Figure S8. SEM images for 1 time, 3 times, 5 times, and 7 times untwisting-twisting processed yarn. (scale bar: 100 μm)

Table S1. Yarn diameters of multiple untwisting-twisting processed yarns in Figure S8.

Twisting-untwisting repeating number	Diameter of LAY (μm)		Average of yarn diameter (μm)
1	Top	135.704	135.0022
		133.919	
		135.693	
		135.740	
	Bottom	133.955	
3	Top	133.919	133.3818
		132.134	
		135.693	
		132.122	
	Bottom	133.041	
5	Top	135.740	136.4514
		137.582	
		132.170	
		139.275	
	Bottom	137.490	
7	Top	133.041	136.1056
		139.275	
		135.740	
		134.098	
	Bottom	138.374	

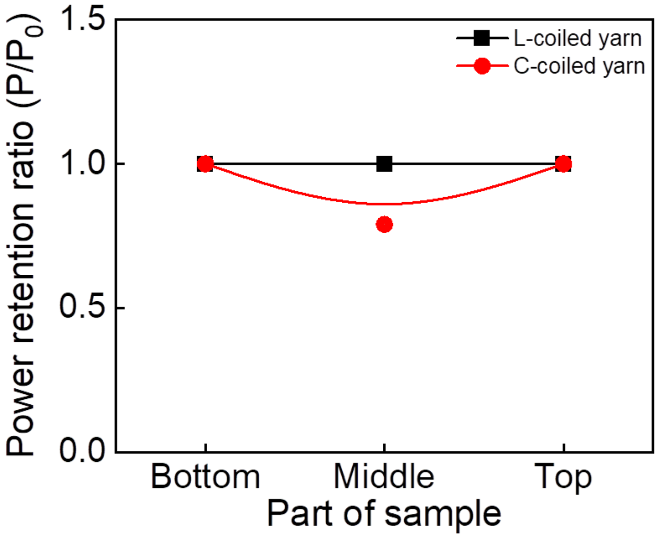

Supplementary Figure S9. Peak power retention ratio of L- (black) and C-coiled yarn (red) along the direction of the coiled yarn. The electrical peak power of the middle and top parts in coiled CNT yarns dividing the bottom end (P_0).

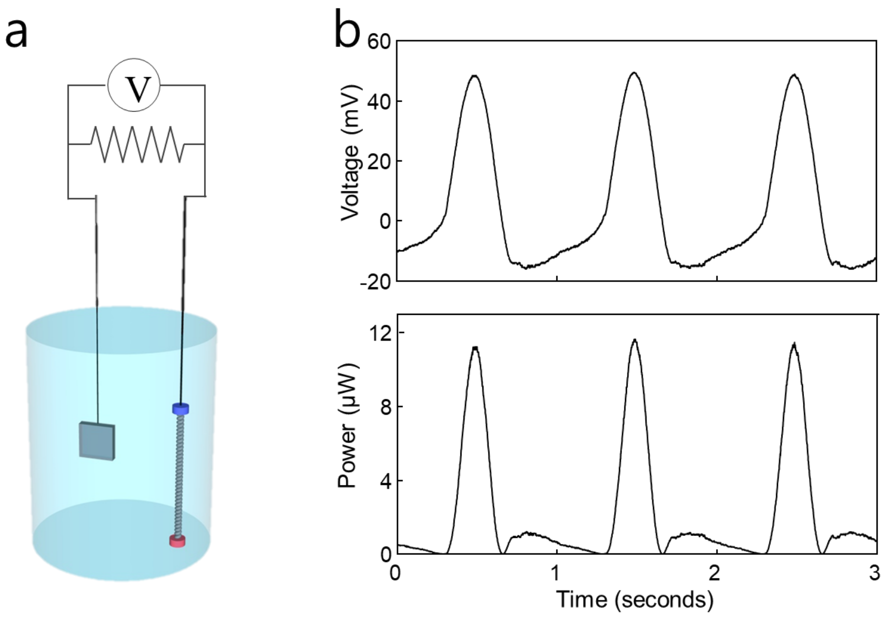

Supplementary Figure S10. a) Two-electrode system for measuring the voltage under variable resistance. b) Real-time open circuit voltage (upper) of the coiled CNT yarn harvester when sinusoidally stretched for three cycles at 1 Hz and resulting electrical power at 210 Ω (bottom).

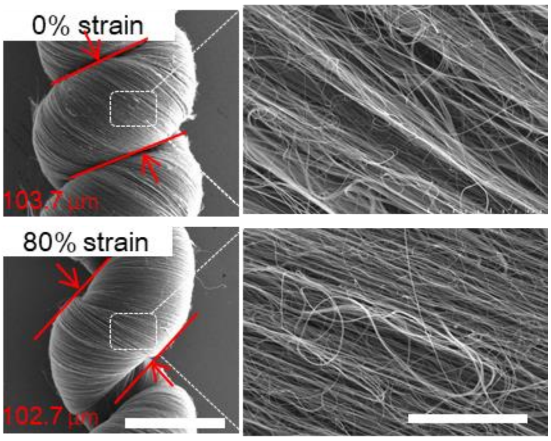

Supplementary Figure S11. SEM images showing C-coiled yarn at 0% and 80% strain (scale bar: 100 µm (left); 5 µm (right)).

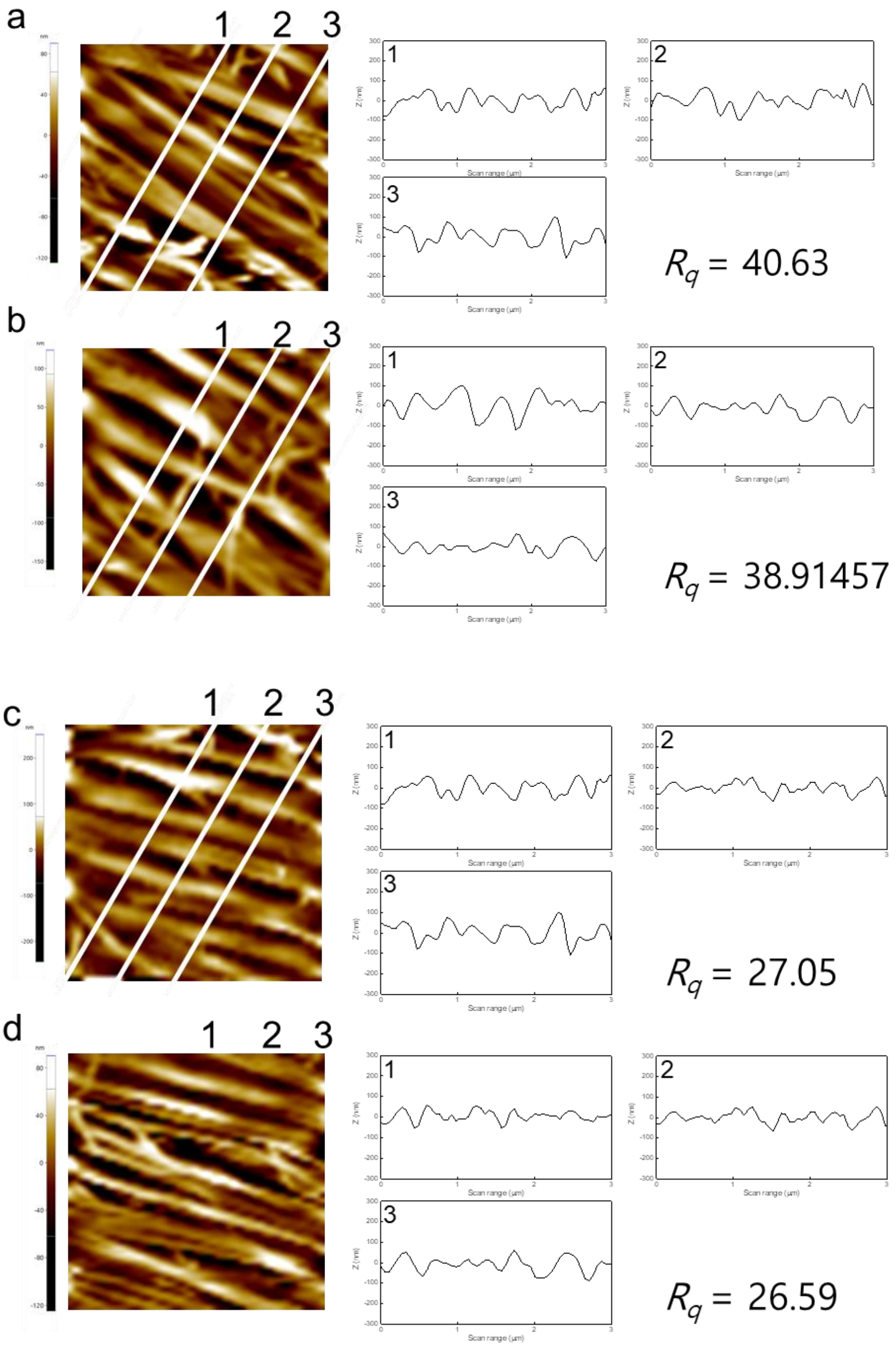

Supplementary Figure S12. AFM images and graphs showing the height difference of the surfaces in L-coiled yarn at a, b) 0% and c, d) 80% strain in the range of 3 × 3 µm. Surface height differences were randomly measured three times along the perpendicular direction of CNT bundles in part of one sample. R_q, the average root mean square (RMS) surface roughness from three extracted locations in one sample (see the experimental section for details).

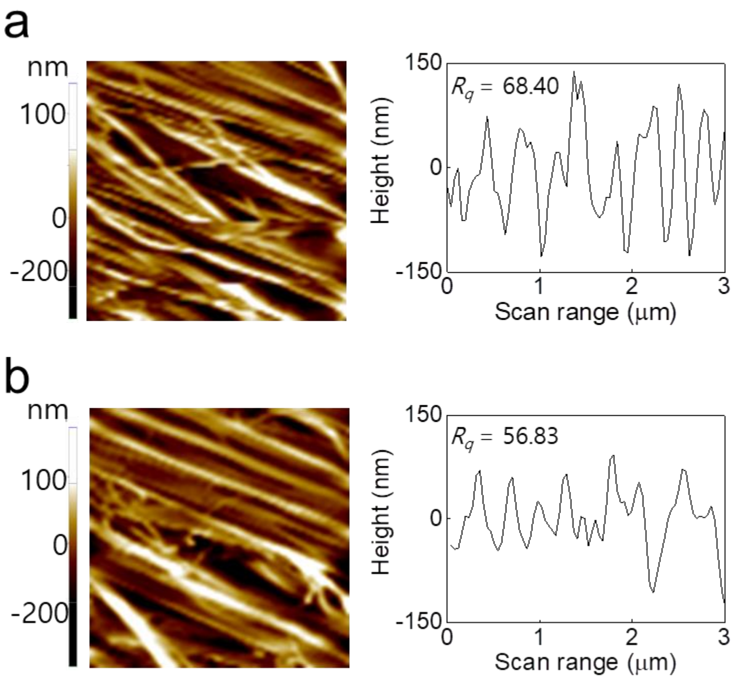

Supplementary Figure S13. Atomic force microscope (AFM) images and graphs showing the height differences of the surfaces in C-coiled yarn at 0% and 80% strain in the range of 3 × 3 μm.

Table S2. Line profile for calculating the average RMS surface roughness (R_q) of the L-coiled yarn shown in Figure S12.

**Number of line profile**	**Measured RMS surface roughness (nm)**			
	0% strain		80% strain	
	a	b	c	d
**1**	38.332067	30.63794	25.55605	25.79753
**2**	39.84702	50.90335	27.42953	26.53166
**3**	43.72159	35.20243	28.16479	27.42953
**Average value**	39.77 ± 0.86		26.82 ± 0.23	

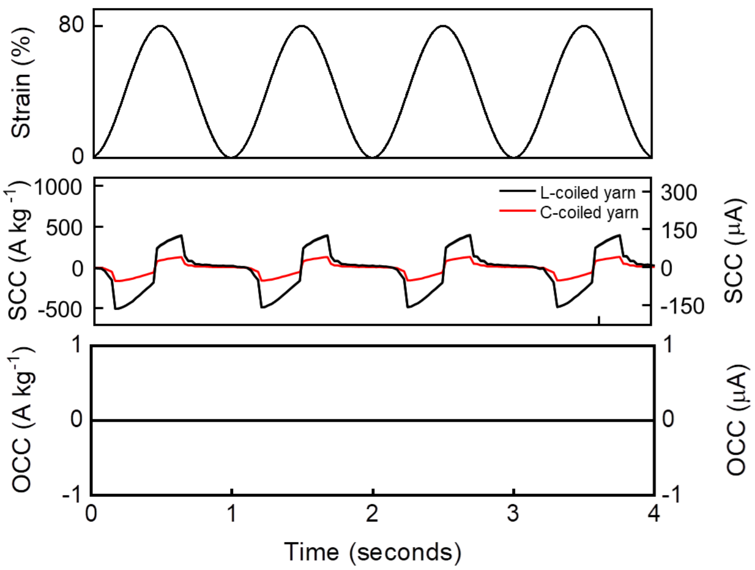

Supplementary Figure S14. Short circuit current (SCC) and open circuit current (OCC) generation in an L-, C-coil yarn harvesters with a spring index of 0.65 when sinusoidally stretched to 80%.

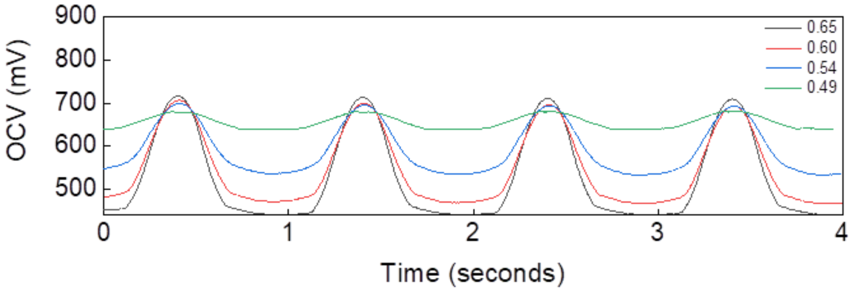

Supplementary Figure S15. Time dependent open-circuit-voltages (OCV) in L-coiled yarn with various spring indices. Variations in OCV of L-coiled yarn with 0.49 (green), 0.54 (blue), 0.60 (red), and 0.65 (black) spring indices generated by 20%, 40%, 50%, and 80% strain.

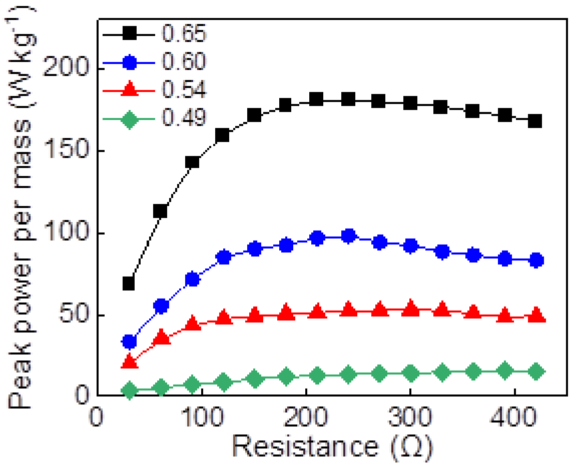

Supplementary Figure S16. Peak power per mass versus load resistance of the L-coiled yarn harvester with a spring index of 0.49, 0.54, 0.60, and 0.65. The matching impedance of each coiled-CNT harvester is 210, 240, 270, and 390 Ω in order of increasing spring index. L-coiled yarn was stretched to 80%, 50%, 40%, and 20% in order of increasing spring index.

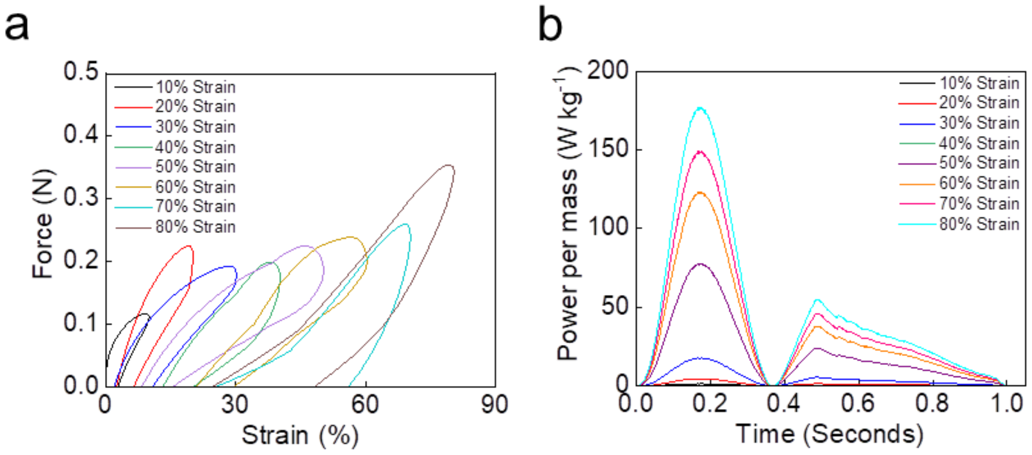

Supplementary Figure S17. a) Required force for various strains from 10% to 80%. The curves indicate mechanical energy. b) The generated power per mass per cycle from 10% to 80%. The area indicates the generated electrical energy per cycle.

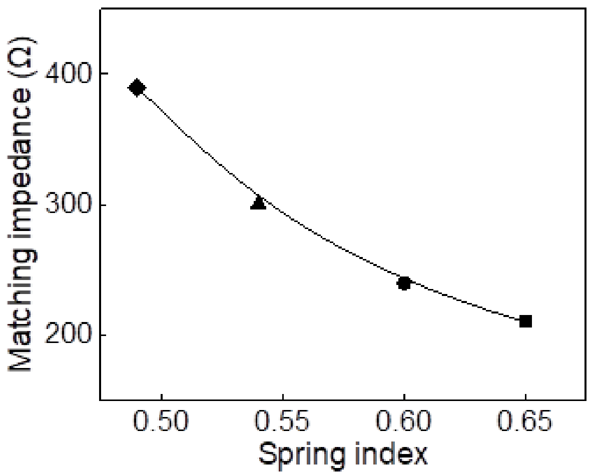

Supplementary Figure S18. Matching impedances of L-coiled yarn harvesters by various spring indexes (square: 0.65, circle: 0.60, triangle: 0.54, diamond: 0.49).

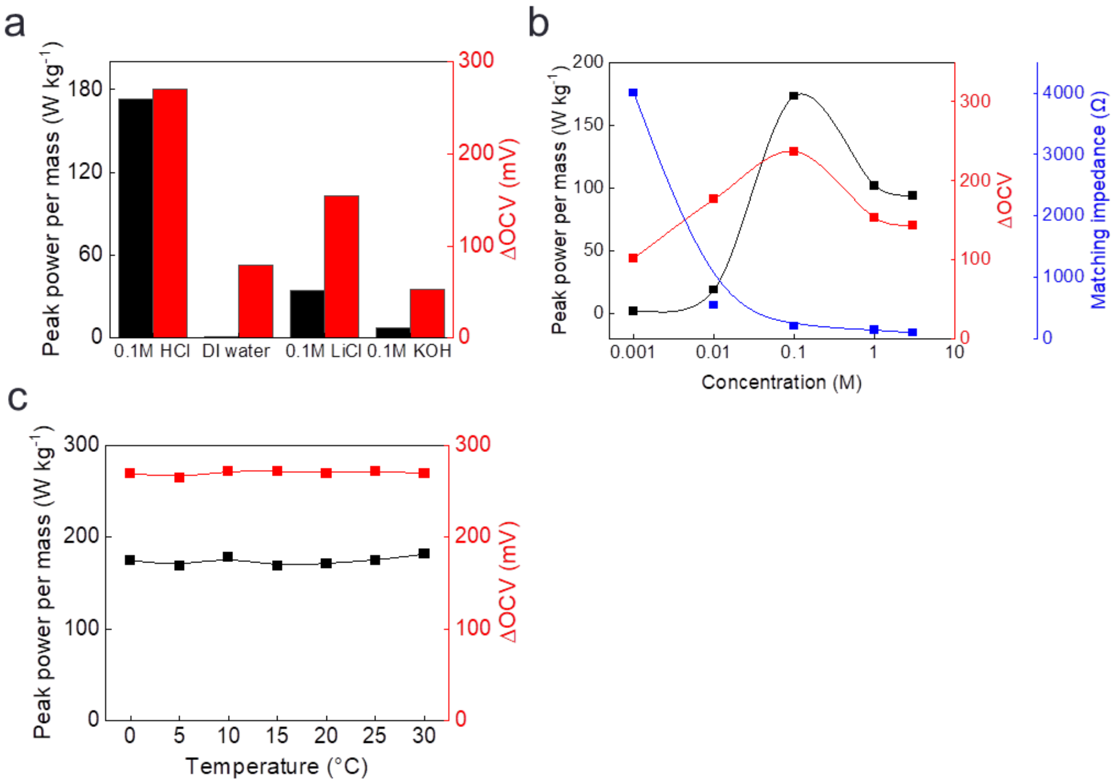

Supplementary Figure S19. The effect of a) different electrolytes (acidic, neutral, and basic solution), b) concentration of HCl, and c) temperature of 0.1M HCl on the performance of a L-coiled yarn harvester with 0.65 spring index during 1Hz stretch to 80% strain.

In order to discuss the effects with different types of electrolytes on the harvester performances, hydrochloric acid, DI water, neutral LiCl and basic KOH with molarity of 0.1 M were investigated (Figure S19a). In DI water, the peak power could not be measured due to the high impedance. The peak power per mass and ΔOCV in acidic HCl electrolyte were higher than others, since decreasing pH increased the intrinsic bias voltage of the harvesters.[14] The relationship between the intrinsic bias voltage of the harvester electrode and ion concentration could be explained by the following Nernst equation:

$$E = E^{o} - \frac{RT}{nF}\log_{10} Q_r$$

where E is the potential of the working electrode, E^o is the standard electrode potential, R is the universal gas constant, T is the temperature in kelvins, n is the number of electrons, F is the Faraday constant, and Q_r is the reaction quotient. Under experimental condition of the room temperature at 25°C and 1 atm, the -59 mV per pH unit is calculated by the Nernst equation. Our L-coiled yarn with 0.65 spring index shows the intrinsic bias voltage dependence on the pH and the slope of -45 mV per pH unit, which is similar with the theoretical value. Increasing the intrinsic bias voltage as decreasing the pH (=increasing the H^+ concentration) indicates charge accumulation on the electrode surfaces by increased ion concentration. Under mechanical deformation, output voltage, peak power, and the matching impedance versus concentration are shown in Figure S19b. The peak power per mass of 168 W kg^{-1} and ΔOCV of 269 mV were maximized at matching impedance of 210 Ω in 0.1 M HCl. Below 0.1 M, the matching impedance was sharply increased with decreased concentration, thus, resulting performances were also decreased. At higher ion concentration than 0.1M, generated voltage and peak power were also decreased. Increase in electrolyte concentration results increase in the number of undissociated salts or less conductive ionic aggregates in solution, which eventually increases the viscosity and decreases the conductivity.[1-2] For this reason, it should be noted that an optimized performance can be achieved with an optimized electrolyte concentration. [14] The effect of temperature on harvesting performance were not significant ranging from 0 °C to 30 °C (Figure S19c). The temperature range was determined in consideration for practical application in the ocean. According to the Nernst equation, the intrinsic bias voltage is affected by changes in temperature, but the temperature range was narrow enough to affect the intrinsic bias voltage, thus, only resulted a slight difference within 4% of average peak power per mass and 2% of average ΔOCV.

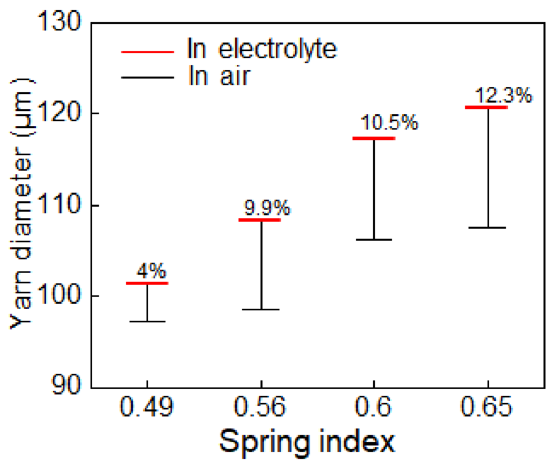

Supplementary Figure S20. Yarn diameter changes caused by the penetration of electrolyte into coiled CNT yarn. Before immersion in electrolyte (black); after immersion in electrolyte (red).

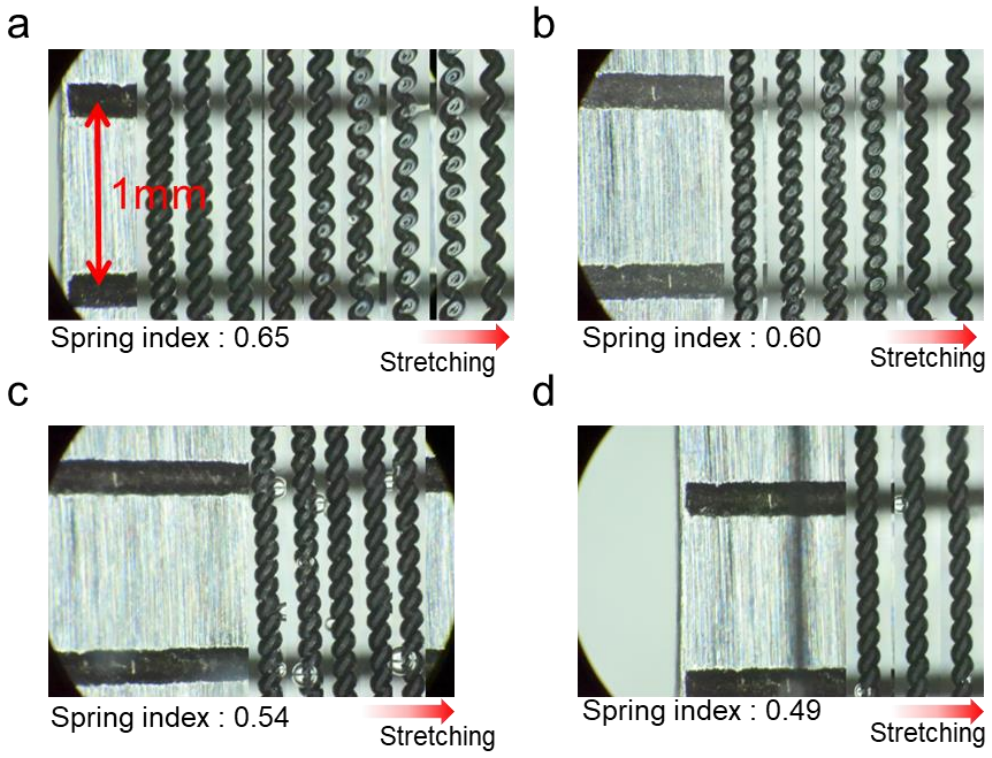

Supplementary Figure S21. Optical microscope images of L-coiled CNT yarn with spring indexes of a) 0.65, b) 0.60, c) 0.54, and d) 0.49. OM images of tensile strain starting at 0% and increasing in 10% increments (scale bar: 1 mm).

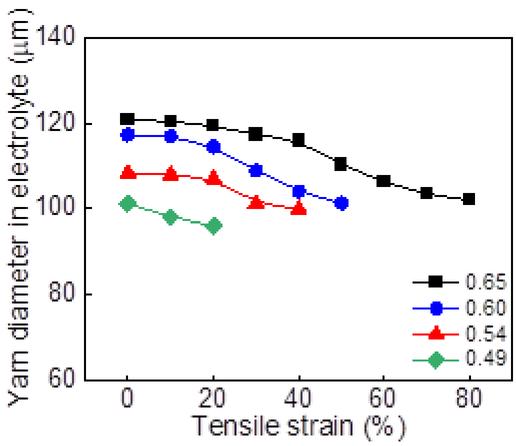

Supplementary Figure S22. Change in yarn diameter by tensile strain during the stretching (from Figure S20) in the electrolyte.

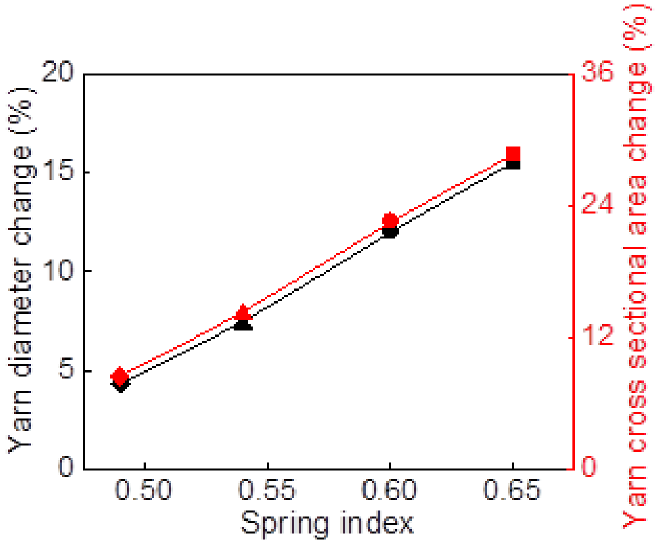

Supplementary Figure S24. Yarn diameter change (black) and yarn cross-sectional area change (red) of L-coiled yarn with spring indexes of 0.49 (diamonds), 0.54 (triangles), 0.60 (circles), and 0.65(squares). The change was calculated at 20%, 40%, 50%, and 80% in order of increasing spring index based on the 0% strain.

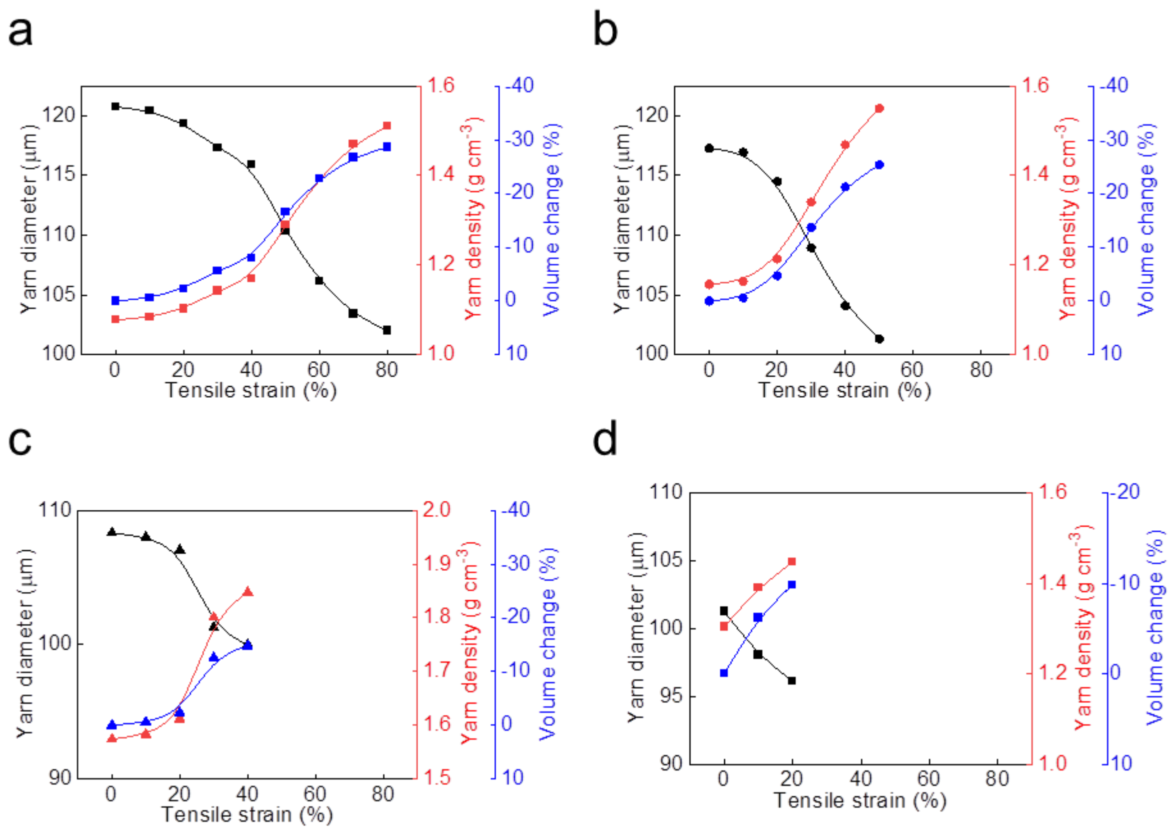

Supplementary Figure S23. Change in diameter (black), density (red), and volume (blue) of L-coiled yarn with spring indices of a) 0.65, b) 0.60, c) 0.54, and d) 0.49.

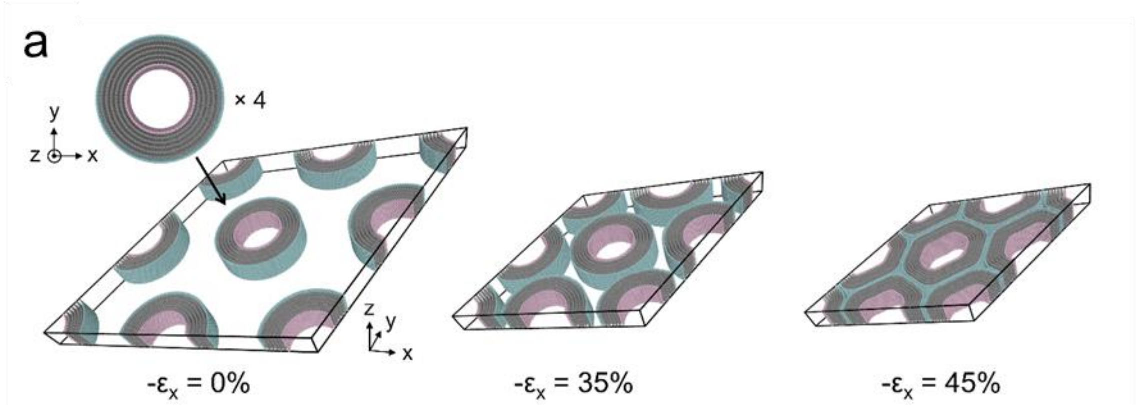

Supplementary Figure S25. Molecular structures of an in-plane hexagonal arrangement of 7-walled-MWCNT and cross-sectional views of the MWCNTs after biaxial compression. The outermost and innermost CNTs are colored cyan and pink, respectively. The interstitial sites between the outermost CNTs are colored blue.

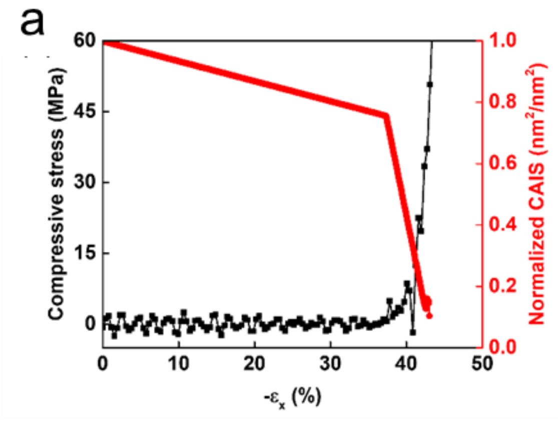

Supplementary Figure S26. Curves of the virial stress in the x-direction and the normalized cross-sectional area of interstitial sites during in-plane compression.

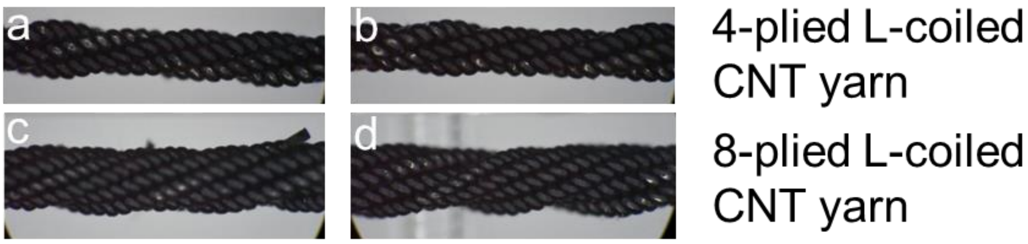

Supplementary Figure S27. Images of a, b) 4-plied L-coiled yarn; c, d) 8-plied L-coiled yarn.

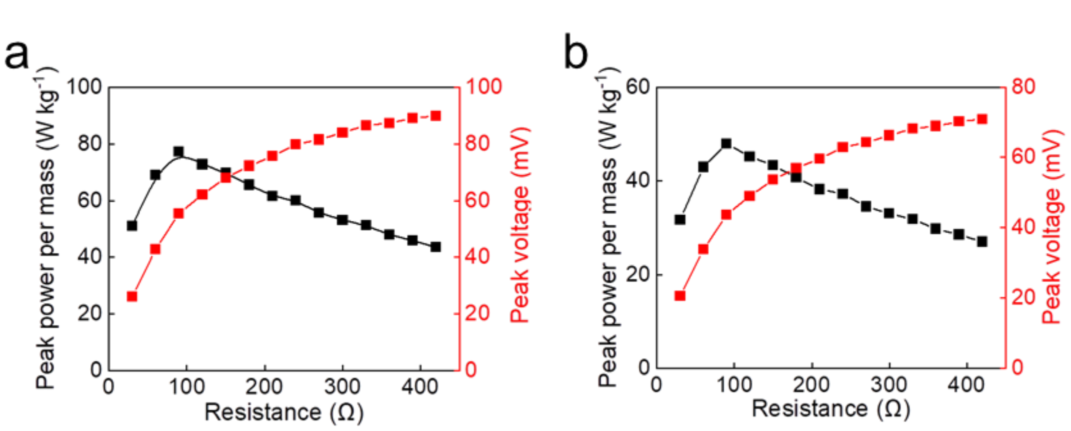

Supplementary Figure S28. Peak power per mass (black) and peak voltage (red) of the 8-plied L-coiled yarn harvester in seawater a) before stabilization and b) after stabilization.

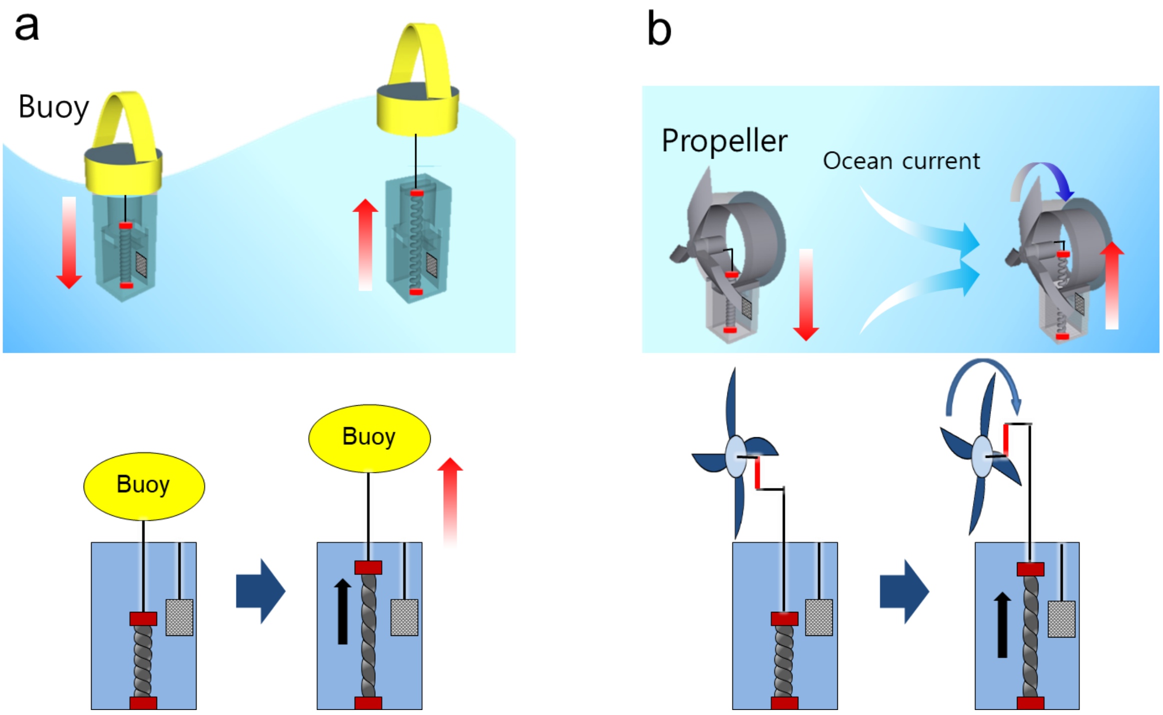

Supplementary Figure S29. Schematic of operation processes for L-coiled yarn harvesters using a) ocean waves and b) currents. Both harvester systems include L-coiled yarn as a working electrode and Pt mesh as a counter electrode. The harvester using ocean waves generates electrical energy with stretching deformation by buoy connected L-coiled yarns. The L-coiled yarn harvester generates electric energy with the propeller connected L-coiled yarn rotation by the ocean current

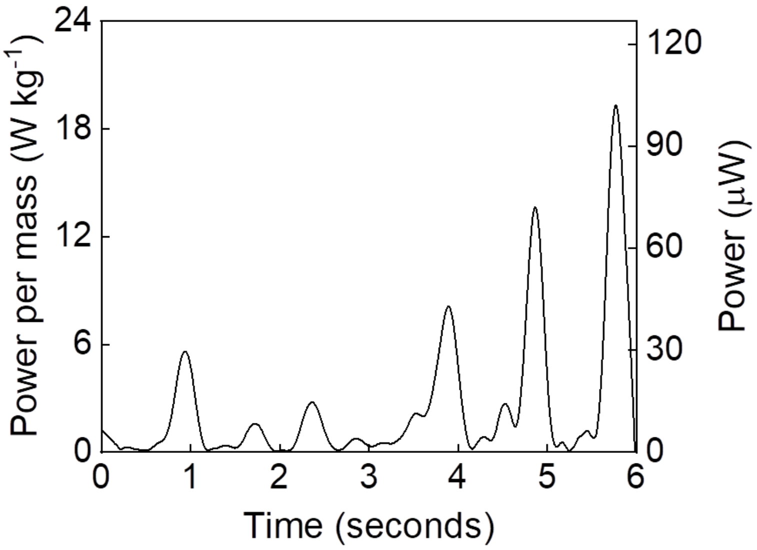

Supplementary Figure S30. Generated electrical power of L-coiled yarn harvester using the structure illustrated in Figure 4c inset (i) and Figure S29a in response to the real waveform at Bangameori Sea off South Korea in August 2022.

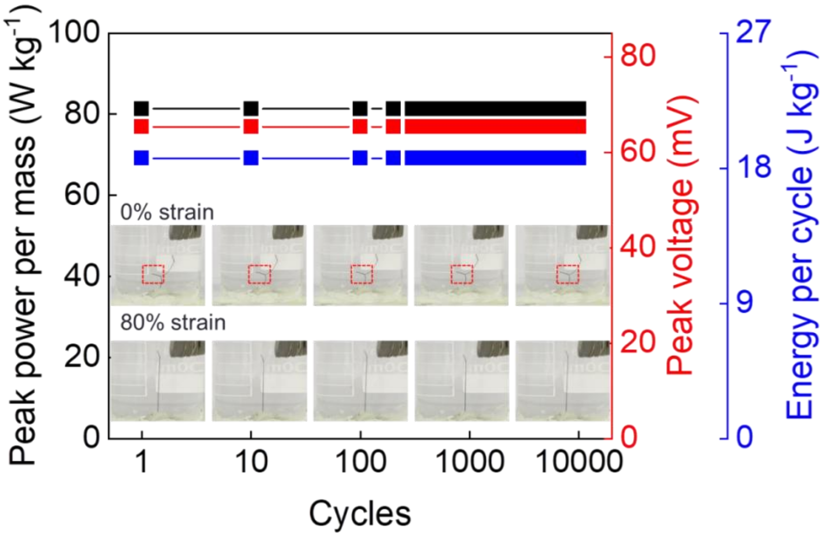

Supplementary Figure S31. Peak power per mass, peak voltage, and electrical energy per cycle during 10,000 stretch-and-release cycles with 80% strain at 1 Hz within 0.6 M NaCl. Inset showing 0% and 80% stretched L-coiled yarn during cycle test.

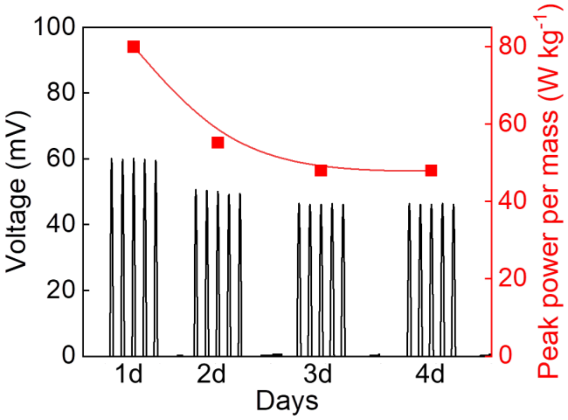

Supplementary Figure S32. Voltage (black) and peak power per mass (red) of the 8-plied L-coiled yarn harvester during the stabilization process. This 4 days of pretest was conducted before the 8 days durability test shown in Figure 4e.

Table S3. Frequency dependent volumetric power of the 8-plied L-coiled yarn harvester when operated within seawater. Other types of various mechanical energy harvesters are included for comparison.

Ref.	Harvester type	Harvester volume (cm^3)	Frequency (Hz)	Volumetric power (mW cm^{-3})
This work	Coiled CNT yarn	0.02545	0.1	1.6
This work	Coiled CNT yarn	0.02545	0.5	6.68
This work	Coiled CNT yarn	0.02545	1	10.45
This work	Coiled CNT yarn	0.02545	3	26.27
[3]	Triboelectric		0.21	0.004
[4]	Triboelectric		0.6	0.0493
[5]	Triboelectric	1767	1	0.0048
[6]	Triboelectric	321.56	1.1	0.02
[7]	Triboelectric		1.2	0.00547
[8]	Triboelectric	3.24	1.5	0.0006917
[9]	Triboelectric		1.8	0.00103
[10]	Triboelectric	179.6	5	0.00712
[11]	Piezoelectric	2.2425	2.5	0.00058
[12]	Piezoelectric	0.000225	33.3	0.00133
[13]	Piezoelectric	0.2313	62.5	0.23
[14]	Liquid-solid triboelectric	0.6174	1	0.16
[15]	Liquid-solid triboelectric	3.24	1.5	0.0072
[16]	Liquid-solid triboelectric	0.768	15	0.00063
[17]	Electrochemical nanogenerator		0.1	0.0487
[17]	Electrochemical nanogenerator		0.21	0.0355
[17]	Electrochemical nanogenerator		1	0.0346

Table S4. Matching impedance dependent volumetric power of the 8-plied L-coiled yarn harvester when operated within seawater. Other types of various mechanical energy harvesters are included for comparison.

Ref.	Harvester type	Harvester volume (cm^3)	Matching impedance (Ω)	Volumetric power ($mW\ cm^{-3}$)
This work	Coiled CNT yarn	0.02545	90	10.45
[18]	Triboelectric		3000000	0.285
[19]	Triboelectric	75	5000000	0.024
[20]	Triboelectric		10000000	0.0049
[21]	Triboelectric	3.96	17400000	0.0024
[22]	Triboelectric	0.03	30000000	9.47
[23]	Triboelectric	14.17	50000000	2.45
[24]	Triboelectric	6.250	100000000	12.2
[25]	Triboelectric	0.088	1000000000	0.21
[26]	Piezoelectric	0.5216	25000	11.14
[13]	Piezoelectric	0.2313	568000	0.23
[11]	Piezoelectric	0.0675	5000000	2.07
[27]	Piezoelectric	1.12	1000000000	5.3E-7
[28]	Liquid-solid triboelectric	0.192	360000	79
[14]	Liquid-solid triboelectric	0.6174	50000000	0.16
[29]	Liquid-solid triboelectric	144	30000000	9.5E-7

Methods

Experimental section

Cylindrical Carbon nanotube sheets fabrication. The CNT forests, which are vertically aligned multi-walled carbon nanotube (MWCNT) nanofibers and a precursor of the CNT yarn, were fabricated by the chemical vapor deposition method. Drawn MWCNT nanofibers from the CNT forest were called CNT sheets, and 4 × 30 cm CNT sheets were stacked side-by-side. Cylindrical CNT sheets were fabricated by rolling both ends of the CNT sheets along the vertical direction. A coiled CNT yarn electrode was fabricated by a motor operating at 1.25 Hz.

Characterization. The structure and apparent properties of twisted, longitudinally aligned, and coiled CNT yarn were observed using field emission scanning electron microscopy (FE-SEM; SU8010, Hitachi Co., Tokyo, Japan). From the measured length, the spring index was calculated as C = R−d/d, where d is the fiber diameter and R is the coil diameter. The bias angle between the twisted yarn and CNTs composing the yarn was also determined via SEM.

Surface analysis. The surface of the coiled CNT yarn was investigated using an atomic force microscope (Park systems XE-70; at 0.3 Hz). From the differences in height, the RMS surface roughness (R_q) was calculated using the following formula:

$$R_\mathrm{q} = \sqrt{\frac{\sum_{i=1}^{n}(Z_i - \bar{Z})^2}{N}} \qquad (1)$$

Electrochemical experiments. The coiled CNT yarn electrode was sinusoidally stretched by the step motor (MD5-HF14). For high frequency (~5 Hz) operation, a servo motor (L7PA002U, FAL01AMK) was used.

The three-electrode system included a coiled CNT yarn electrode as a working electrode, Pt mesh / MWNT bucky paper as a counter electrode, and Ag / AgCl as a reference electrode. In the three-electrode system, the cyclic voltammetry and open circuit voltage were measured. For measuring the power, the coiled CNT yarn harvester included the coiled CNT yarn electrode as the working electrode and Pt mesh / MWNT bucky paper as the counter electrode. Experiments were carried out by either discontinuously stretching by 10% or continuously stretching up to the maximum tensile length of coiled CNT yarn. All measurements were conducted in 0.1 M HCl, except for application tests. In application tests, seawater from the Gyeonpo Sea off South Korea was used as the electrolyte.

An electrochemical analysis device (Zive SM6, WonA Tech.) was used for assessing cyclic voltammetry and galvanostatics. The scan rate and range were set at 50 mVs^{-1} and 400-700 mV, respectively. The voltage was measured by a wireless memory hilogger (LR8450-01, LR8533, HIOKI) for application tests.

Mechanical tests. The mechanical energy of the L-coiled CNT yarn when sinusoidally stretched from 10% to 80% with 10% intervals at 1 Hz was calculated from the stress-strain curve measured by the fatigue tester (MTS ACUMEN 3, MTS, USA).

Capacitance and power calculation. From the cyclic voltammetry curves, the gravimetric capacitance (C) was calculated as follows:

$$C = I \,/\, (dV \,/\, dt) \,/\, m \qquad (2)$$

where I is the average discharge current, dV/dt is the scan rate, and m is the mass of the coiled CNT yarn.

The peak power (P) was calculated as follows:

$$P = V^2 / R / m \tag{3}$$

where V is the peak voltage, R is the impedance matched resistance, and m is the mass of the coiled CNT yarn.

Additional twist insertion calculation. ΔT was calculated in the electrolyte using the following formula:

$$\Delta T = \frac{N\Delta L}{l^2} \tag{4}$$

where ΔT is the increased twist density with increasing coil length (ΔL), N is the number of coils, and l is the fiber length.

Molecular dynamics (MD) simulation conditions. A molecular structure consisting of 7-walled-MWCNTs with an outer diameter of 8.95 nm was prepared to be close to that in experimental conditions. The molecular structure contained four identical MWCNT strands arranged hexagonally in the x–y plane of the triclinic unit cell. Periodic boundary conditions were applied along three axes to represent hexagonally packed MWCNT bundles. The initial density of the modeled MWCNT yarn unit cell (MC) was determined to be 0.7 g/cc, following their extraction from a sparsely arranged MWCNT forest.[30] The detailed geometry of the MC is presented in Table 1. The MC was equilibrated for 300 ps under a canonical (NVT) ensemble thermostat at 300 K.[31] During the equilibration period, an AIREBO-M (adaptive intermolecular reactive empirical bond order modified by Morse potential) was used[32] to describe interatomic interactions between carbon atoms under high external pressure.

Table 1. Detailed geometry of the MC

d_o (nm)	d_i (nm)	$L_x = L_y$ (nm)	L_z (nm)	ρ (g/cm^3)
8.95	4.93	28.06	2.56	0.70

When a mechanical load was applied to the coiled yarn harvester, the radial strain on the cross-section of each MWCNT bundle dominantly controlled the microstructural change.[33] To characterize such structural state changes through the MD simulations, quasi-static biaxial compression in the x–y plane was induced under a strain rate of 10^7 /s at 0.1 K. In-plane compression was applied until the engineering strain in the x- and y-directions reached $-\varepsilon_x = -\varepsilon_y = 50\%$, resulting in the radial buckling of closely packed MWCNTs.[34] The region in the vicinity of the MWCNT surfaces was assumed to be filled with electrolyte, which affected the double-layer capacitance. The cross-sectional area of interstitial sites was calculated during biaxial compression by excluding the accessible surface of MWCNTs[35] from the unit cell. Simultaneously, the virial stress tensor ($\boldsymbol{\sigma}$) exerted on the faces of unit cells was expressed as follows.[36]

$$\boldsymbol{\sigma}_{ij} = \frac{1}{\Omega}\left(-\sum_i^N m_i(\mathbf{v}_i\mathbf{v}_i^T) + \frac{1}{2}\sum_i^N \sum_{j\neq i}^N \mathbf{r}_{ij}\mathbf{F}_{ij}\right) \quad (5)$$

where Ω is the volume of the domain; m_i and $\mathbf{v}_i$ are the mass and velocity of the ith particle in the domain, respectively; and $\mathbf{r}_{ij}$ and $\mathbf{F}_{ij}$ are the distance and interaction between i^{th} and j^{th} particles, respectively. Because the in-plane compression was performed at approximately zero temperature, Eqn. 6 is explicitly expressed by the potential term, which makes the virial stress equivalent to the continuum Cauchy stress for the system considered.[37]

8-plied L-coiled yarn fabrication. Eight L-coiled yarns were prepared individually via the application of a load. Afterwards, the top and bottom of the aligned eight L-coiled yarns were grouped together, and their rotation was fixed at the bottom. Then, a twisting force was applied to the top to make 8-plied L-coiled yarn.

References

[1] V. Chaudov, J. Jacquemin, F. Tran-Van, M. Deschamps and F. Ghamouss, *Pure and Applied Chemistry* **2019**, *91*, 1361–1381.

[2] J. Jiang, H. Ji, P. Chen, C. Ouyang, X. Niu, H. Li and L. Wang, *Jounal of Power Sources* **2022**, *527*, 231193.

[3] L. Liu, X. Yang, L. Zhao, H. Hong, H. Cui, J. Duan, Q. Yang and Q. Tang, *ACS nano* **2021**, *15*, 9412-9421.

[4] W. Zhong, L. Xu, X. Yang, W. Tang, J. Shao, B. Chen and Z. L. Wang, *Nanoscale* **2019**, *11*, 7199-7208.

[5] X. Liang, T. Jiang, G. Liu, Y. Feng, C. Zhang and Z. L. Wang, *Energy & Environmental Science* **2020**, *13*, 277-285.

[6] J. An, Z. M. Wang, T. Jiang, X. Liang and Z. L. Wang, *Advanced Functional Materials* **2019**, *29*, 1904867.

[7] J. Ren, C. Gao, J. An, Q. Liu, J. Wang, T. Jiang and Z. L. Wang, *Advanced Materials Technologies* **2021**, *6*, 2100359.

[8] Y. Xu, W. Yang, X. Lu, Y. Yang, J. Li, J. Wen, T. Cheng and Z. L. Wang, *ACS nano* **2021**, *15*, 16368-16375.

[9] M. Xu, T. Zhao, C. Wang, S. L. Zhang, Z. Li, X. Pan and Z. L. Wang, *ACS nano* **2019**, *13*, 1932-1939.

[10] L. Xu, T. Jiang, P. Lin, J. J. Shao, C. He, W. Zhong, X. Y. Chen and Z. L. Wang, *ACS nano* **2018**, *12*, 1849-1858.

[11] S. An, H. S. Jo, G. Li, E. Samuel, S. S. Yoon and A. L. Yarin, *Advanced Functional Materials* **2020**, *30*, 2001150.

[12] G.-J. Lee, M.-K. Lee, J.-J. Park, D. Y. Hyeon, C. K. Jeong and K.-I. Park, *ACS applied materials & interfaces* **2019**, *11*, 37920-37926.

[13] X. Huang, Q. Qin, X. Wang, H. Xiang, J. Zheng, Y. Lu, C. Lv, K. Wu, L. Yan and N. Wang, *ACS nano* **2021**, *15*, 19783-19792.

[14] C. Ye, D. Liu, X. Peng, Y. Jiang, R. Cheng, C. Ning, F. Sheng, Y. Zhang, K. Dong and Z. L. Wang, *ACS nano* **2021**, *15*, 18172-18181.

[15] X. Wei, Z. Zhao, C. Zhang, W. Yuan, Z. Wu, J. Wang and Z. L. Wang, *ACS nano* **2021**, *15*, 13200-13208.

[16] W. Zhong, L. Xu, F. Zhan, H. Wang, F. Wang and Z. L. Wang, *Acs Nano* **2020**, *14*, 10510-10517.

[17] M. Zohair, K. Moyer, J. Eaves-Rathert, C. Meng, J. Waugh and C. L. Pint, *ACS nano* **2020**, *14*, 2308-2315.

[18] B. Cheng, Q. Xu, Y. Ding, S. Bai, X. Jia, Y. Yu, J. Wen and Y. Qin, *Nature communications* **2021**, *12*, 1-8.

[19] R. Zhang, C. Dahlström, H. Zou, J. Jonzon, M. Hummelgård, J. Örtegren, N. Blomquist, Y. Yang, H. Andersson and M. Olsen, *Advanced Materials* **2020**, *32*, 2002824.

[20] H. Ryu, H.-m. Park, M.-K. Kim, B. Kim, H. S. Myoung, T. Y. Kim, H.-J. Yoon, S. S. Kwak, J. Kim and T. H. Hwang, *Nature communications* **2021**, *12*, 1-9.
[21] C. Dong, A. Leber, T. Das Gupta, R. Chandran, M. Volpi, Y. Qu, T. Nguyen-Dang, N. Bartolomei, W. Yan and F. Sorin, *Nature communications* **2020**, *11*, 1-9.
[22] S. Chun, C. Pang and S. B. Cho, *Advanced Materials* **2020**, *32*, 1905539.
[23] L. Long, W. Liu, Z. Wang, W. He, G. Li, Q. Tang, H. Guo, X. Pu, Y. Liu and C. Hu, *Nature communications* **2021**, *12*, 1-10.
[24] J. Jeong, S. Jeon, X. Ma, Y. W. Kwon, D. M. Shin and S. W. Hong, *Advanced Materials* **2021**, *33*, 2102530.
[25] L. Ma, R. Wu, S. Liu, A. Patil, H. Gong, J. Yi, F. Sheng, Y. Zhang, J. Wang and J. Wang, *Advanced materials* **2020**, *32*, 2003897.
[26] X. Yuan, X. Gao, J. Yang, X. Shen, Z. Li, S. You, Z. Wang and S. Dong, *Energy & Environmental Science* **2020**, *13*, 152-161.
[27] J. Sun, H. Guo, J. Ribera, C. Wu, K. Tu, M. Binelli, G. Panzarasa, F. W. Schwarze, Z. L. Wang and I. Burgert, *ACS nano* **2020**, *14*, 14665-14674.
[28] Y. Chen, B. Xie, J. Long, Y. Kuang, X. Chen, M. Hou, J. Gao, S. Zhou, B. Fan and Y. He, *Advanced Materials* **2021**, *33*, 2104290.
[29] J. Nie, Z. Wang, Z. Ren, S. Li, X. Chen and Z. Lin Wang, *Nature communications* **2019**, *10*, 1-10.
[30] a) K. R. Atkinson, S. C. Hawkins, C. Huynh, C. Skourtis, J. Dai, M. Zhang, S. Fang, A. A. Zakhidov, S. B. Lee and A. E. Aliev, *Physica B: Condensed Matter* **2007**, *394*, 339-343; b) M. Yu, H. H. Funke, J. L. Falconer and R. D. Noble, *Nano Letters* **2009**, *9*, 225-229.
[31] H. C. Andersen, *The Journal of chemical physics* **1980**, *72*, 2384-2393.
[32] T. C. O'Connor, J. Andzelm and M. O. Robbins, *The Journal of chemical physics* **2015**, *142*, 024903.
[33] J. Foroughi, G. M. Spinks, G. G. Wallace, J. Oh, M. E. Kozlov, S. Fang, T. Mirfakhrai, J. D. Madden, M. K. Shin and S. J. Kim, *Science* **2011**, *334*, 494-497.
[34] a) B. Goh, K. J. Kim, C.-L. Park, E. S. Kim, S. H. Kim and J. Choi, *Carbon* **2021**, *184*, 452-462; b) B. Goh, K. J. Kim, E. S. Kim, S. H. Kim and J. Choi, *Applied Surface Science* **2021**, *561*, 150075.
[35] M. L. Connolly, *Science* **1983**, *221*, 709-713.
[36] D. M. Heyes, *Physical Review B* **1994**, *49*, 755.
[37] a) A. K. Subramaniyan and C. Sun, *International Journal of Solids and Structures* **2008**, *45*, 4340-4346; b) J. Choi, H. Shin, S. Yang and M. Cho, *Composite Structures* **2015**, *119*, 365-376.
